# R-R Interval Histogram-Based Deep Learning for 3-Class Atrial Fibrillation Screening in Garment-Type Wearable Holter Electrocardiogram Monitoring: Algorithm Development and Validation Study

**DOI:** 10.2196/91960

**Published:** 2026-07-24

**Authors:** Tomoaki Nakano, Keita Okayama, Masanao Yasuoka, Hironori Shigeta, Akito Kawamura, Takayuki Sekihara, Kentaro Ozu, Takafumi Oka, Shigeto Seno, Yasushi Sakata

**Affiliations:** 1 Department of Cardiovascular Medicine, Graduate School of Medicine, The University of Osaka, 2-2, Yamadaoka, Suita, Osaka, 565-0871, Japan, 81 06-6879-3632; 2 Department of Bioinformatic Engineering, Graduate School of Information Science and Technology, The University of Osaka, Suita, Osaka, Japan

**Keywords:** atrial fibrillation, deep neural network, paroxysmal atrial fibrillation, long-term Holter electrocardiogram, R-R interval

## Abstract

**Background:**

Long-term garment-type wearable Holter electrocardiographic (ECG) monitoring is frequently affected by noise contamination, which complicates automated atrial fibrillation (AF) detection in real-world recordings. Although deep learning has shown high performance for AF detection, relatively few studies have evaluated explicit strategies for handling noise-included wearable ECG data. An alternative representation using the R-R interval (RRI) time series may reduce the dependence on waveform morphology and provide an alternative pathway for AF screening in noisy recordings.

**Objective:**

This study aimed to develop and evaluate a 3-class, noise-aware RRI-based AF screening framework that explicitly separated AF, non-AF, and uninterpretable noise windows, and to assess the impact of analysis window length on model performance.

**Methods:**

Single-lead garment-type wearable Holter ECG data from 117 patients at the University of Osaka Hospital were analyzed after exclusion of patients with documented atrial tachycardia, flutter, or paced rhythm according to the predefined task definition. R-peaks were automatically detected, and the resulting RRI segments were converted into 2D histogram images, with time on the x-axis and RRI-derived heart rate on the y-axis, for 1.5-, 3-, and 6-minute windows. A ResNet-34–based 2D convolutional neural network was trained for 3-class classification. Model performance was evaluated using 5-fold interpatient cross-validation on the institutional dataset and independent external testing on the MIT-BIH (Massachusetts Institute of Technology–Beth Israel Hospital) AF Database (AFDB). In the external validation, atrial flutter–annotated intervals were excluded to match the training task definition. Patient-level AF burden was evaluated by comparing reference AF burden with model-estimated AF burden using Pearson and Spearman correlation coefficients, and linear regression.

**Results:**

Of 129 monitored patients between March 1, 2023, and November 20, 2025, 117 were analyzed. In the internal validation, the 3-class model (non-AF, AF, and noise) showed similarly high performance for the 1.5- and 3-minute windows, both with an accuracy of 96.6%. In independent external validation, the 3-minute window showed numerically the highest overall performance (accuracy: 97.3%; AF sensitivity: 96.9%; and AF specificity: 97.7%), although the differences across window lengths were modest. At the patient level, AF burden correlation was high across all window lengths, with Pearson *r* of 0.995, 0.991, and 0.989 and Spearman ρ of 0.988, 0.982, and 0.979 for the 1.5-, 3-, and 6-minute models, respectively.

**Conclusions:**

The RRI-based 2D convolutional neural network achieved high AF classification accuracy and strong patient-level correlation with reference AF burden. Using RRI features and a 3-class framework, which explicitly separated noise from AF and non-AF rhythms, a 3-minute RRI window provided a favorable balance of performance for AF screening in a garment-type Holter ECG.

## Introduction

Atrial fibrillation (AF) is a common arrhythmia and a major cause of cardioembolic stroke [1,2]. Early and accurate detection of paroxysmal AF (PAF), which may manifest asymptomatically or transiently, is important for timely anticoagulation and rhythm control strategies. Given the unpredictable onset of PAF, long-term Holter electrocardiographic (ECG) monitoring has been extensively used to capture AF episodes [3-7]. Garment-type wearable Holter ECG devices, which integrate electrodes into shirts or belts, have garnered attention for their ability to facilitate long-term continuous recording during daily activities with minimal burden on the wearer [8-10].

In garment-type wearable Holter ECG monitoring, the characteristics of noise contamination differ from those of conventional adhesive or patch-type recordings. As the garment can be removed and reworn, recordings may contain not only intermittent contact artifacts during wear, caused by body motion, variable electrode contact, and changes in skin-electrode impedance, but also off-body periods, signal loss intervals, and segments acquired during unstable reattachment [9,11-13]. These heterogeneous segments are ultimately included in the long-term recordings submitted for clinical review, making manual analysis by clinical technologists or physicians time-consuming and labor-intensive [3,14,15]. Therefore, the practical challenge in diagnostic support using automated analysis is not limited to improving AF detection accuracy in relatively low-noise ECG segments but also includes avoiding forced classification into AF or non-AF in windows in which rhythm interpretation is unreliable. Recent studies, including the PhysioNet or Computing in Cardiology Challenge 2017, have emphasized the importance of AF classification from noisy single-lead ECG recordings and have advanced algorithm development in this setting [7,16,17]. However, corresponding evidence in long-term garment-type wearable Holter ECG remains limited.

Automatic ECG analysis using AI, particularly deep learning (DL), has recently emerged as a promising approach to address these challenges. Although DL architectures that directly process raw ECG waveforms, including 1D convolutional neural networks (1D-CNNs) [14-18], have demonstrated high performance, their performance may deteriorate in the presence of waveform noise in wearable recordings. In contrast, approaches based on the R-R interval (RRI) focus on the beat-to-beat irregularity, which is fundamental to AF and may be relatively tolerant of waveform contamination [19-22]. Moreover, converting the RRI time series into 2D images allows AF rhythm characteristics to be represented as structured visual patterns, making this approach highly compatible with analyses based on 2D convolutional neural networks (2D-CNNs) [23-25]. In noise-controlled patch-type Holter recordings, such RRI-based 2D-CNN approaches have achieved high accuracy with short segment windows of approximately 90 beats [4,24].

However, a suitable RRI-based DL framework for noise-prone garment-type monitoring remains unclear. In particular, it is uncertain how analysis window length influences diagnostic performance when analyzable rhythm portions and uninterpretable artifact-contaminated portions coexist within long-term recordings. Therefore, this study aimed to develop and evaluate an RRI histogram-based 3-class AF screening framework for wearable Holter ECG recordings, in which visually uninterpretable or unreliable segments were treated as an independent noise class, separate from AF and non-AF rhythms. By examining multiple analysis window lengths, we sought to identify an approach that could support efficient and reliable AF screening in real-world wearable monitoring.

## Methods

### Study Design

#### Task Definition

This study aimed to develop a machine learning framework for AF screening based on RRI irregularity while explicitly separating uninterpretable segments as noise. Atrial tachycardia (AT) and atrial flutter (AFL) typically exhibit regular RRIs and lack the irregular characteristics of AF, thus representing distinct physiological rhythmic phenotypes [26]. Therefore, AT/AFL episodes were excluded from both the training and validation datasets to preserve the clinical coherence of the task rather than to optimize model performance. The diagnosis and characterization of AT/AFL constitute a separate clinical challenge and are considered beyond the scope of this analysis.

In addition, this study was designed to systematically evaluate the impact of window length as part of a sensitivity analysis of the performance of the RRI-based AF detection model in the context of long-term garment-type wearable Holter ECG monitoring, which is susceptible to noise contamination. Therefore, noise was treated as a distinct label to reflect real-world data quality and enable the model to explicitly separate uninterpretable segments from AF and non-AF rhythms. Three window lengths were examined: 1.5-, 3-, and 6-minute windows.

#### Study Population

The study population comprised patients who underwent monitoring using a wearable Holter ECG device (PS201-01; Mitsufuji) at the University of Osaka Hospital between March 1, 2023, and November 20, 2025. Examinations were performed to evaluate suspected or known arrhythmias or symptoms such as palpitations.

#### Ethical Considerations

This study was approved by the Ethics Committee of the University of Osaka Hospital on March 1, 2023 (approval 22449) and was conducted in accordance with the Declaration of Helsinki. Written informed consent was obtained from all participants prior to study enrollment. All study data were deidentified before analysis to protect participant privacy and confidentiality. No financial compensation or other incentives were provided to the participants. No images in the manuscript or supplementary material contain information that could identify individual participants.

### ECG Recording and Data Collection

**Figure 1.**
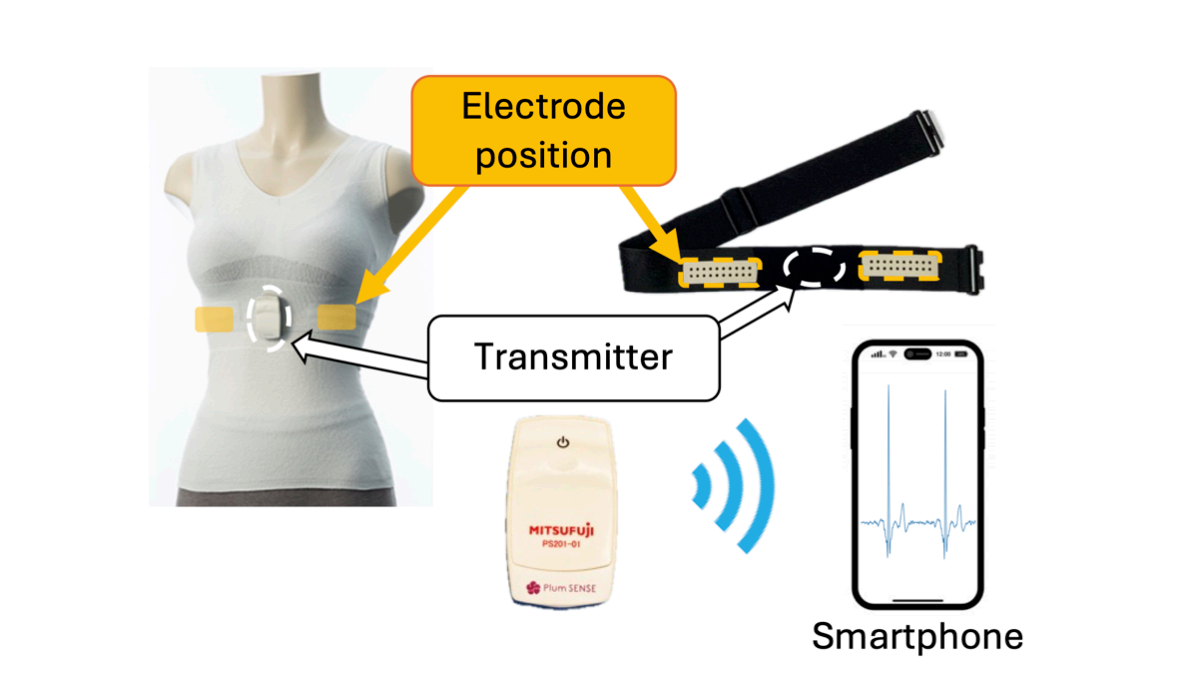
Configuration of the garment-type wearable Holter ECG system. The shirt-type garment (left) uses electrodes incorporating silver fiber AGposs, whereas the belt-type garment (right) uses electrodes incorporating both AGposs and the silver-containing paste DuraQ. The orange frames indicate the electrode positions. The transmitter was attached within the white dotted-line area and transmitted electrocardiogram data via Bluetooth to a smartphone app, where the signals were stored.

ECG data were acquired using a PS201-01 wearable Holter ECG device [10] with either shirt-type or belt-type garment electrodes (Figure 1).

#### Device Configuration

The PS201-01 system uses silver fibers (AGposs; Mitsufuji) as the electrode material. The shirt-type garment used AGposs alone, whereas the belt-type garment combined AGposs with electrodes made from a silver-containing paste (DuraQ; Sumitomo Bakelite). Both garments share an equivalent electrode configuration, enabling the acquisition of a single-lead ECG corresponding to lead I of a standard 12-lead ECG.

#### Data Acquisition Specifications

The ECG waveforms acquired using the PS201-01 device were transmitted via Bluetooth to a dedicated smartphone app (wearable Holter ECG management; Mitsufuji) and stored in the Medical Waveform Format Encoding Rules format. The device is a class II long-term ECG recording system approved under the Japanese Pharmaceuticals and Medical Devices Act. The ECG signal was directly sampled by the built-in analog-to-digital converter at 250 Hz and stored at the same sampling frequency without downsampling; no analog-to-digital converter (ADC) internal digital filter or digital low-pass filter was applied before storage. According to manufacturer-provided circuit simulation data, the analog front end was designed to provide band limiting for antialiasing before ADC sampling. The relative gain compared with the passband was −2 dB at 100 Hz, −4.5 dB at 125 Hz, −10 dB at 250 Hz, −20 dB at 700 Hz, and −33 dB at 1000 Hz; the −3 dB, −20 dB, and −40 dB frequencies were approximately 105 Hz, 700 Hz, and 1.25 kHz, respectively. The sampling frequency of 250 Hz was identical to that used in the MIT-BIH AFDB.

### Data Preprocessing and Annotation

For model development, the RRI segments were extracted from raw ECG waveforms and annotated by a cardiologist.

#### RRI Data Generation

For ECG data stored in the Medical Waveform Format Encoding format, an automatic R-peak detection algorithm based on the Hamilton method [27], as implemented in the Python BioSPPy library, was applied to generate the RRI time series data. No subsample interpolation, dedicated artifact-rejection procedure, or additional peak refinement technique was applied after automatic detection. Baseline correction was performed using a bandpass filter as a part of the preprocessing pipeline. In noise-labeled segments, R-peak detection was not uniformly reliable and could result in excessive false detections, absent or insufficient detections, or temporally heterogeneous detection patterns, depending on the type and severity of artifact. As the accuracy of R-peak detection critically influences model performance in RRI-based AF diagnosis, this step serves as the foundation of the entire analysis workflow. To support the practical validity of this preprocessing step under 250 Hz sampling, we performed a supplementary quality assessment of R-peak detection described in the following section.

#### Quality Assessment of R-Peak Detection

As a supplementary quality check of the preprocessing pipeline, we performed a manual review of randomly sampled 30-second ECG segments from the institutional dataset used for model development and internal validation. This assessment was performed to evaluate the practical reliability of the Hamilton-based R-peak detection step in the same data source used for training and testing, but it was not used to select, exclude, or reassign segments for model training or testing. All windows were handled according to the predefined label aggregation rules and patient-level cross-validation splits, regardless of the quality assessment results. The review set consisted of 100 pure non-AF segments, 100 pure AF segments, 50 pure noise segments, and 50 noise-containing segments. A cardiologist visually reviewed each segment to determine whether reliable reference R-peaks could be identified. A segment was defined as *annotatable* when R-peaks could be reliably confirmed for at least 20 of the 30 seconds. For annotatable segments, automatic R-peak detection was graded as *acceptable* when >90% of automatically detected peaks appropriately corresponded to visually identified R waves, having *minor errors* when the proportion was >70% to ≤90%, and *unreliable* otherwise. For noise and noise-containing segments, readability was also recorded.

#### Annotation Procedure

The generated RRI plots and the corresponding raw ECG waveforms were visualized using an in-house ECG annotation tool. A cardiologist reviewed the continuous ECG recordings in chronological order and assigned labels at 10-second intervals, taking the surrounding rhythm context into account. AF was defined as a rhythm showing irregular RRIs without discernible P waves, occupying more than half of a given 10-second interval. When multiple rhythms occurred within a single interval, the rhythm occupying the majority of the interval was assigned as the representative label. Thus, transitions between rhythms were handled by majority labeling within each 10-second interval.

A cardiologist classified the data into 3 classes:

- Non-AF: normal sinus rhythm and non-AF arrhythmias, excluding AT/AFL
- AF: atrial fibrillation
- Noise: a 10-second interval in which reliable rhythm interpretation was not possible because of severe artifact, unstable electrode contact, signal loss, or periods during which the garment was not worn

The following ECG patterns were excluded from the analysis: regular supraventricular tachycardia (AT/AFL) and segments containing paced waveforms.

Annotations were created independently of model training and evaluation. To further assess annotation robustness, supplementary intrarater and independent interrater agreement analyses were performed, as described in the “Annotation Agreement Assessment” section.

#### Annotation Agreement Assessment

We performed a supplementary annotation verification analysis using randomly sampled ECG segments from the institutional dataset used for model development and internal validation. A custom program was developed to randomly sample segments and display the corresponding ECG data for manual review. Using this procedure, 150 segments were randomly selected, comprising 50 non-AF, 50 AF, and 50 noise segments according to the original study annotations. The sampled segments were then reassessed in 2 ways: first, by the cardiologist who had created the original annotations, using the dedicated review program, and second, by an additional independent cardiologist. Agreement of each reassessment with the original study annotations was evaluated using percentage agreement and Cohen κ. This supplementary analysis was designed to examine the robustness of the original annotations and was not used to select, exclude, relabel, or reassign segments for model training or testing. The original study annotations remained the reference labels for model development and evaluation, and all windows were handled according to the predefined label aggregation rules and patient-level cross-validation splits.

#### Patient-Level Rhythm Burden Assessment

To characterize the rhythm composition of the institutional cohort, patient-level AF burden was calculated using the original 10-second annotations. AF burden was defined as the proportion of AF-labeled intervals among analyzable intervals after excluding noise-labeled intervals from the denominator. The distribution of AF burden across patients was summarized to assess whether the cohort contained a broad range of PAF patterns or was dominated by sustained AF and non-AF recordings.

To address the concern that the non-AF class might consist predominantly of clean sinus rhythm, we performed a rudimentary ectopic burden estimate in patients with an AF burden of 0%. This subgroup was selected because all analyzable intervals in these patients were annotated as non-AF, allowing assessment of ectopy in non-AF recordings without contamination by AF-to-non-AF transition periods. As exhaustive ectopic beat annotation across the full dataset was not feasible and could be affected by noise, representative ECG segments were sampled for manual review. For each eligible patient, six 3-minute segments were selected from the available analyzable non-AF recordings, and ectopic beats were visually identified. Ectopic burden was calculated as the proportion of ectopic beats among all visually assessable beats in the reviewed segments. Patients were categorized according to estimated ectopic burden as <1%, 1% to <5%, 5% to <10%, or ≥10%. This analysis was intended to provide a descriptive estimate of ectopy within the non-AF class rather than a comprehensive beat-level annotation of the entire dataset.

### Feature Extraction and Training Data Generation

Two-dimensional feature images used as inputs to the DL model were generated from the RRI time series data to construct the training and evaluation datasets.

#### Segmentation of RRI Data

The RRI time series data were segmented into fixed-length analysis windows. Window lengths of 1.5, 3, and 6 minutes were used, and model performance was evaluated for each setting. These window lengths were selected to cover clinically and methodologically relevant time scales. A 1.5-minute window approximately corresponds to 90 beats at a heart rate of 60 beats per minute, which is similar to the segment length used in prior Lorenz/Poincaré plot–based AF detection studies [23-25]. A 6-minute window was included because 6 minutes have been used as a clinically relevant threshold in studies of atrial high-rate episodes [28,29]. A 3-minute window was evaluated as an intermediate setting to assess the trade-off between temporal resolution with shorter windows and classification stability with longer windows.

#### Data Augmentation (Sliding Window)

**Figure 2.**
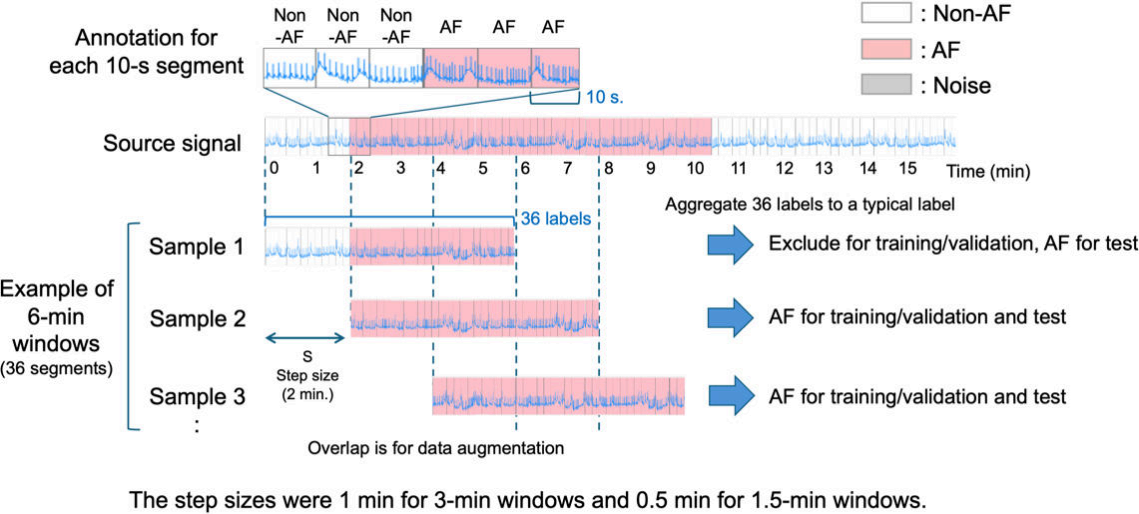
Schematic overview of sliding window data augmentation and segment-level label assignment used for model development. Ground-truth annotations were provided at 10-s intervals and aggregated to a single segment label (36 labels for a 6-min window; 18 labels for a 3-min window; and 9 labels for a 1.5-min window). Segments containing both atrial fibrillation (AF) and non-AF labels were excluded from the training or validation dataset but handled according to the test time rule (The detailed label aggregation rules are provided in the “Label Aggregation Rules” subsection).

To improve model generalization and increase the volume of training data, a sliding window approach was applied to all window length settings (Figure 2). To standardize the augmentation strategy across models while preserving a similar relative degree of overlap, the step size was set to one-third of the target window length. Accordingly, step sizes of 30 seconds, 1 minute, and 2 minutes were used for the 1.5-minute, 3-minute, and 6-minute models, respectively. Cross-validation was performed per patient to prevent interpatient data leakage caused by the sliding window procedure. Thus, overlapping windows derived from the same patient were contained within the same data split and were not shared across training, validation, and test sets. No additional procedure was applied to reduce correlation among overlapping samples within the same split; rather, the sliding window approach was used as an intended augmentation strategy within the training framework.

#### Conversion to 2D Images (Histogram Processing)

Rather than using the raw 1D RRI sequence directly as input, each segment was transformed into a 2D histogram image to represent rhythm irregularity in a structured form. AF is characterized not only by variability in interval length itself but also by the temporal pattern and density distribution of beat-to-beat fluctuations. A 2D representation was therefore adopted to capture both the overall distribution of interval-derived values and their sequential variation, conceptually similar to Lorenz/Poincaré-based rhythm analysis [23-25]. In addition, this representation allowed noisy or visually uninterpretable segments to appear as scattered and unstable patterns. Histogram binning aggregates neighboring values and may reduce the influence of minor timing fluctuations or isolated R-peak detection errors. Heat maps were generated as 2D histograms from each RRI segment using the window-level R-peak positions on the x-axis and heart rate derived from the RRI on the y-axis. Specifically, heart rate was calculated as 60,000 divided by the RRI in milliseconds, and the histogram was computed as a count-based density map without additional count normalization. Thus, the present input representation was a time–heart rate density histogram rather than a recurrence plot or a ΔRRI map. The y-axis represented RRI-derived heart rate and was predefined to range from 30 to 400 beats per minute, uniformly divided into 200 bins for all window lengths. Thus, RRIs longer than 2.0 seconds, corresponding to heart rates below 30 beats per minute, and extremely short RRIs below 150 milliseconds, corresponding to heart rates above 400 beats per minute, were outside the predefined heart rate range of the histogram representation. These out-of-range intervals were not encoded as separate overflow bins or dedicated features in the model input. For the x-axis, the number of bins was scaled according to segment duration to maintain comparable temporal density across models: 25 bins for 1.5-minute windows, 50 bins for 3-minute windows, and 100 bins for 6-minute windows. Accordingly, the resulting histogram image sizes were 25×200, 50×200, and 100×200 bins, respectively. Histogram counts were used as pixel intensities, and no additional smoothing or interpolation was applied before input into the 2D-CNN. An enlarged representative RRI histogram image is provided in Figure S1 in Multimedia Appendix 1 to improve visual interpretability of the histogram format. As noise-labeled segments were defined by visually uninterpretable or nondiagnostic ECG quality rather than by a single R-peak detection failure mode, their RRI-derived histograms were heterogeneous. Representative noise-labeled examples included excessive false R-peak detections, absent or insufficient detections, and temporally heterogeneous artifact patterns within the same window. Real ECG waveforms with automatically detected R-peak positions and the corresponding RRI-derived heart rate histograms are shown in Figure S2 in Multimedia Appendix 1.

#### Label Aggregation Rules

**Figure 3.**
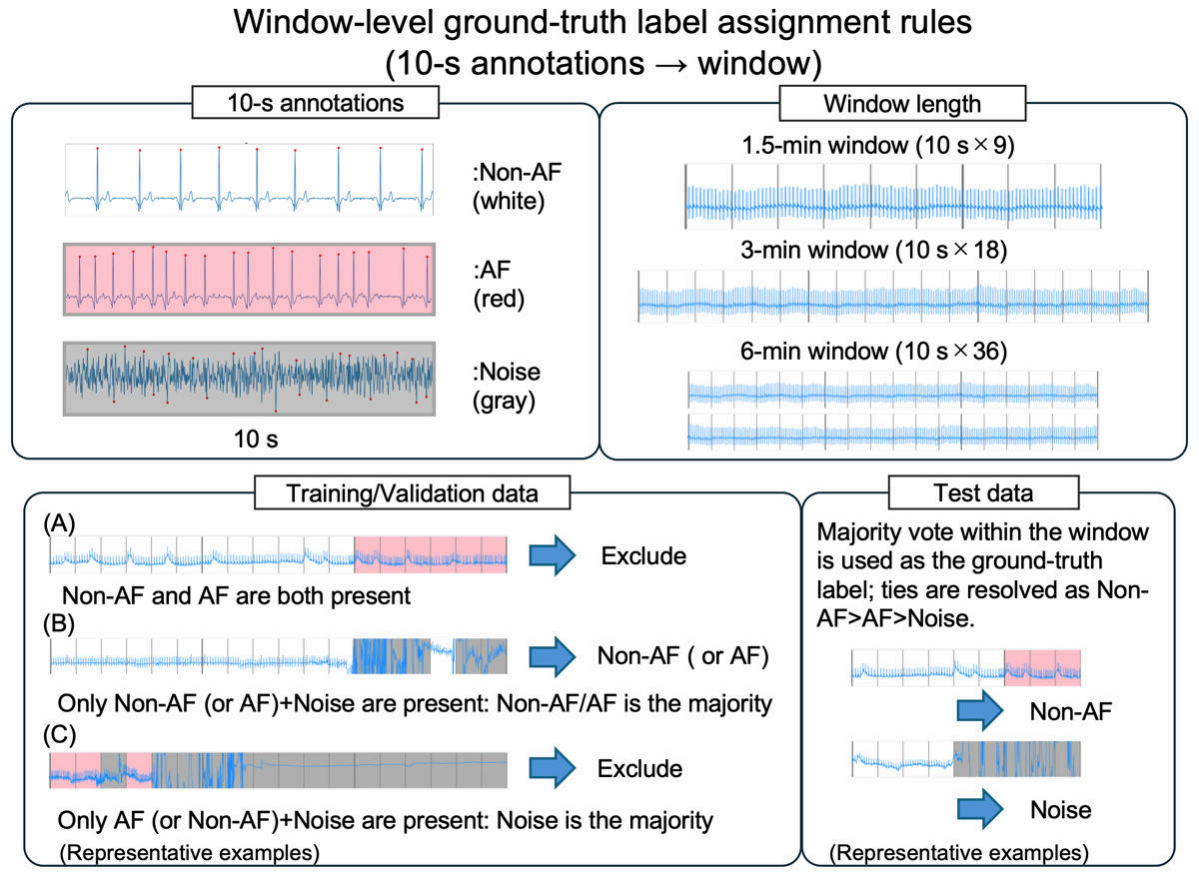
Window-level ground-truth label assignment rules for the 3-class classification task. Annotations were assigned in 10-s intervals. Consequently, each segment corresponded to 9 labels for a 1.5-min window, 18 labels for a 3-min window, and 36 labels for a 6-min window. The figure illustrates the rules for handling windows containing multiple labels, together with representative electrocardiogram (ECG) examples for both training/validation and test data. Ten-second labels are indicated by background color: red for atrial fibrillation (AF), gray for noise, and white for non-AF. The Noise example shown in the figure represents an actual artifact-contaminated waveform recorded using the garment-type wearable Holter ECG device, not simulated Gaussian noise.

As annotations were assigned at 10-second intervals, each segment corresponded to 9 labels for a 1.5-minute window, 18 labels for a 3-minute window, and 36 labels for a 6-minute window. The final window label was determined using separate rule sets for the training/validation and test datasets, as illustrated in Figure 3.

### Training or Validation Data

The data used for training or validation were handled according to the following rules:

- For supervised training, windows containing both AF and non-AF labels were excluded because assigning a single representative label to such windows would introduce ambiguous supervisory signals. This rule was adopted to preserve clear label definitions during model training rather than to deny the existence of rhythm transitions in real-world recordings. This approach may reduce the representation of transitional or mixed rhythm segments in the training dataset and is therefore considered a limitation of the present framework.
- For supervised training, windows containing both AF and non-AF labels were excluded because assigning a single representative label to such windows would introduce ambiguous supervisory signals. This rule was adopted to preserve clear label definitions during model training rather than to deny the existence of rhythm transitions in real-world recordings. This approach may reduce the representation of transitional or mixed rhythm segments in the training dataset and is therefore considered a limitation of the present framework.

### Test Data

The data used for test were handled according to the following rules:

- The most frequently used label within a window (majority vote) was used as the reference label.
- In the event of a tie, the reference label was determined using a predefined priority order (non-AF > AF > noise) solely to ensure deterministic label assignment. This priority order was an operational convention for uncommon edge cases and was not intended to represent a clinically motivated hierarchy among rhythm classes.

### DL Model and Validation Protocol

A supervised DL model was constructed to classify the generated 2D RRI images into non-AF, AF, and noise classes.

#### DL Model

We adopted ResNet-34, a 2D-CNN architecture with residual learning that enables stable training in deep networks and has shown strong performance in image recognition [30]. ResNet-34 was selected as a practical compromise between representational capacity and model complexity within the ResNet family. In the context of the present wearable ECG dataset, we considered shallower models potentially insufficient to capture the diversity of rhythm patterns, whereas deeper models could increase the risk of overfitting without a clear advantage. To accommodate the present input representation, the initial convolutional layer was modified to accept single-channel inputs, and the final pooling layer was replaced with an adaptive global average pooling layer. As a result, the model could accept the native histogram image sizes (25×200, 50×200, and 100×200) without resizing to a fixed square resolution. The model was initialized from scratch, and no pretrained weights were used. No additional systematic hyperparameter optimization was performed beyond the predefined training settings and early stopping. The model was trained to classify the histogram images derived from the RRI segments into 3 classes: non-AF, AF, and noise. Training was performed using the Adam optimizer (learning rate 1×10⁻⁴), with cross-entropy loss as the objective function.

#### Internal Validation

Internal validation was performed using 5-fold cross-validation on an institutional dataset. To avoid leakage of patient information between the training and test sets, we used an interpatient paradigm, splitting data at the patient level. Patients were randomly divided into 5 mutually exclusive groups based on their identifiers. For each fold, approximately one-fifth of the patients were assigned to the test set, and the remaining four-fifths were used for model development, with the development set further divided into training and validation subsets at a 9:1 ratio. The training split within the nontest patients was also performed at the patient level by random sampling without stratification; therefore, all sliding window samples derived from a given patient were assigned exclusively to either the training or validation subset. No explicit balancing was applied for patient characteristics, such as age, AF burden, or signal quality across folds. The training and evaluation were repeated for each fold using the same procedure. Given the relatively limited size of the institutional dataset, early stopping was used as the primary measure to reduce overfitting. Specifically, training was halted if the validation loss did not improve for 5 consecutive epochs. No additional formal hyperparameter search was performed. As an exploratory post hoc subgroup analysis by garment type, we evaluated the 3-minute model using the same 5-fold cross-validation splits as those used in the primary internal validation. As the shirt-type subgroup had a limited sample size and included few AF cases, separate garment-specific model training was not performed. Instead, in each fold, the model was trained using mixed shirt- and belt-type recordings, and pooled out-of-fold predictions from the corresponding test folds were stratified by garment type.

#### External Validation

To evaluate the generalization performance of the predefined model framework, external validation was conducted using the MIT-BIH AFDB, a widely used benchmark for AF detection. The AFDB was completely independent of the institutional dataset and was used exclusively as an external test dataset; it was not used for model training, validation, or tuning. The AFDB contains 25 two-channel ECG recordings, each approximately 10 hours in duration and sampled at 250 Hz. Two recordings (00735 and 03665) were excluded due to missing signals. As the sampling frequency of 250 Hz matched that of the wearable Holter device, resampling was not required. To match the single-lead configuration of the wearable device, only the channel labeled ECG1 was used for all AFDB recordings; the 2 channels were neither averaged nor randomly selected.

The AFDB contains annotations comprising 4 rhythm categories: atrial fibrillation (AFIB), atrial flutter (AFL), junctional rhythm (JR), and normal rhythm (NR). Each event was annotated with a sample-level onset position and corresponding label. For comparison with the proposed method, these annotations were converted into 10-second interval labels. When multiple labels occurred within a 10-second interval, the label with the longest duration was assigned as the representative label.

As AT/AFL episodes were excluded from the institutional training and validation datasets, AFL-annotated intervals were excluded from the primary external validation analysis to ensure consistency between the training task definition and the external test framework. Intervals corresponding to AFIB were mapped to the AF class, whereas those corresponding to JR and NR were mapped to the non-AF class. No additional supraventricular rhythm categories were separately annotated in the AFDB; therefore, no further rhythm-specific remapping was performed. As the database lacked explicit noise labels, intervals containing visually apparent noise were newly annotated and assigned to the noise class. Noise was defined as a visually uninterpretable ECG segment in which reliable rhythm assessment was not possible because of artifact. These annotations were confirmed by 2 cardiologists. As a secondary analysis, we also evaluated an alternative framework in which AFL intervals were mapped to the AF class, reflecting a broader clinical grouping of atrial tachyarrhythmias.

For external validation, the final model was retrained using the full institutional development dataset with the same predefined architecture and training settings used in the internal validation. A 9:1 training:validation split was used for early stopping only. This approach was chosen so that all available institutional development data contributed to the final model before testing on the completely independent AFDB. The same early stopping strategy was used, in which training was halted if the validation loss did not improve for 5 consecutive epochs. This model was then used to segment the RRI data from the AFDB into segments of each window length, convert them into 2D images, and generate predictions. The same core preprocessing pipeline was used for both the institutional data and AFDB, including R-peak detection from the full recording and generation of window-level RRI histogram images. The main AFDB-specific preprocessing steps were exclusion of nontarget intervals before window generation and remapping of the public annotation labels as described earlier. The reference label for each window was defined as the label of the longest rhythmic region in that window.

To evaluate the clinical relevance of aggregated model outputs across an entire recording, we assessed the correlation between the reference AF burden and the model-estimated AF burden on a per-patient basis. In the external validation implementation, AFL-annotated intervals were first excluded in accordance with the primary task definition. The reference AF burden was then calculated for each patient, based on the AFDB annotations, as the proportion of AF-labeled 10-second intervals among analyzable intervals after excluding noise-labeled intervals from the denominator. Model-estimated AF burden was similarly calculated as the proportion of windows classified as AF among windows classified as either AF or non-AF, thereby excluding windows classified as noise from the denominator. Mixed-rhythm windows were handled according to the predefined label assignment and window aggregation rules and were not subjected to an additional exclusion step during patient-level burden calculation.

### Accuracy Evaluation and Statistical Analysis

#### Diagnostic Performance Metrics

Model performance was evaluated at the window level using overall accuracy and class-wise sensitivity, specificity, positive predictive value (PPV), negative predictive value (NPV), *F*_1_-score, and area under the receiver operating characteristic curve (AUROC) for the 3 classes (AF, non-AF, and noise). Overall accuracy was defined as the proportion of correctly classified windows among all evaluated windows. For each class, sensitivity, specificity, PPV, NPV, and *F*_1_-score were calculated using a one-versus-rest framework, in which the target class was treated as positive and the other 2 classes were combined as negative. For the internal validation, these metrics were calculated separately for each of the 5 cross-validation test folds and then summarized across folds. Macroaveraged *F*_1_-score and macroaveraged AUROC were additionally calculated to provide an overall assessment that was less affected by class imbalance. AUROC was calculated using a one-versus-rest approach for each class.

For AF burden, the association between reference and model prediction was assessed at the patient level. Pearson correlation coefficient was used as the primary measure to assess the linear association between the reference and model-estimated AF burden values. As AF burden may show a skewed distribution, Spearman rank correlation coefficient was additionally calculated as a supplementary nonparametric measure. Linear regression was also performed to characterize the relationship between the 2 methods.

#### Statistical Analysis Environment

All analyses were performed using Python 3.8.8 on the Windows 11 platform. The computational environment comprised a 3.6 GHz 12-core Intel Core i7 CPU, 32 GB DDR5-4800 memory, and an ASUS GeForce RTX 3080 Ti GPU (12 GB). The ECG annotation was performed using a custom-built ECG annotation tool (GitHub). AI-assisted tools were used only for code drafting and refactoring. Specifically, ChatGPT (OpenAI; during 2025, JST) and GitHub Copilot (GitHub; during 2025, JST) assisted in the Python boilerplate generation and readability improvements. All scripts were reviewed, tested, and finalized by the authors, who took full responsibility for the analyses. No confidential or patient-identifiable information was entered into the tool.

## Results

### Study Population and Baseline Characteristics

Between March 1, 2023, and November 20, 2025, 129 patients underwent monitoring with a single-lead garment-type wearable Holter ECG device. Of these, 12/129 (9.3%) patients with documented AT/AFL or paced rhythm were excluded according to the predefined task definition. Consequently, 117/129 (90.7%) patients were included in model development and internal validation. Their baseline characteristics are summarized in Table 1.

**Table 1.** Baseline characteristics of the institutional cohort included in model development and internal validation at the University of Osaka Hospital (N=117).

Characteristic	Value
Age (y), mean (SD)	69.6 (12.5)
Sex (male), n (%)	77 (65.8)
Height (cm), mean (SD)	163.5 (9.4)
Weight (kg), mean (SD)	65.2 (14.9)
BMI (kg/m²), mean (SD)	24.2 (4.0)
History of AF^a^, n (%)	99 (84.6)

a AF: atrial fibrillation.

**Figure 4.**
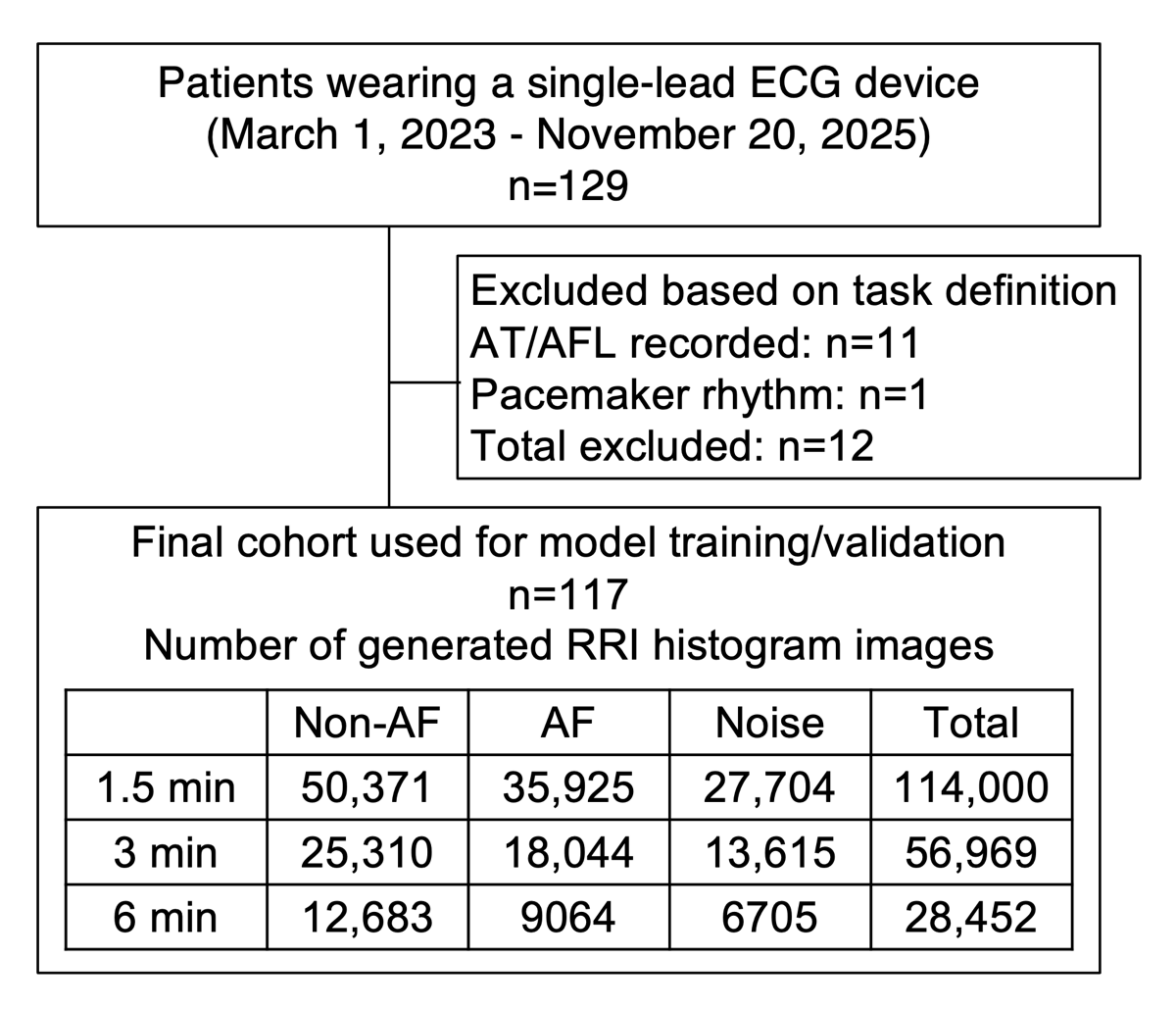
Study cohort selection and dataset generation for model development and validation in garment-type wearable Holter ECG monitoring at the University of Osaka Hospital between March 1, 2023, and November 20, 2025. Flow diagram showing patient selection from the institutional cohort, exclusion of atrial tachycardia/atrial flutter (AT/AFL) and pacemaker cases according to the predefined task definition, and the number of generated R-R interval (RRI) histogram images for model development and internal validation. The number of generated RRI histogram images represents unique window-level samples before sliding window augmentation and corresponds to the total number of test windows across the 5 cross-validation folds. ECG: electrocardiogram.

The institutional dataset comprised a total of 2850 hours of ECG recordings from 117 patients. After segmentation and label aggregation, the number of unique window-level samples before data augmentation for the 1.5-, 3-, and 6-minute settings was 114,000, 56,969, and 28,452, respectively (Figure 4). Across all window length settings, non-AF windows were the most frequent class, whereas noise windows were the least frequent, indicating class imbalance in the generated datasets.

### Rhythm and Signal Quality Characteristics in the Institutional Cohort

To characterize the rhythm composition of the institutional cohort, patient-level AF burden was calculated from the original 10-second annotations as the proportion of AF-labeled intervals among analyzable intervals. The AF burden distribution was bimodal: 53 (45.3%) patients had an AF burden of 0%, whereas 49 (41.9%) patients had an AF burden of ≥80%; patients with intermediate AF burden were relatively infrequent (Table S1 in Multimedia Appendix 2).

In the rudimentary semiquantitative review of ectopic burden among 53 patients with an AF burden of 0%, estimated ectopic burden was <1% in 36 (67.9%) patients, 1% to <5% in 8 (15.1%) patients, 5% to <10% in 3 (5.7%) patients, and ≥10% in 6 (11.3%) patients. This review suggested that the non-AF recordings were not limited to exceptionally clean sinus rhythm. The distribution is summarized in Table S2 in Multimedia Appendix 2.

Noise-labeled intervals were commonly observed in the institutional recordings. The total number of noise-labeled 10-second intervals was 249,285, corresponding to approximately 692.5 hours of noise-labeled recording. At the patient level, the median noise burden was 17.6% (IQR 7%‐36%), and only 2 of 117 patients had a noise burden of 0%. These findings indicate that uninterpretable or unreliable segments were commonly present in long-term garment-type wearable Holter ECG recordings. The detailed distributions of noise burden are provided in Table S3 in Multimedia Appendix 2.

### Quality Assessment of R-Peak Detection

In the supplementary review of 300 randomly sampled 30-second ECG segments, 238 (79.3%) were considered annotatable. All pure non-AF segments and all pure AF segments were annotatable, and all these were graded as acceptable. Almost all pure noise segments (49/50, 98%) were judged nonannotatable and unreadable. Among noise-containing segments, 37 (74%) of 50 were annotatable and readable. Across the 238 annotatable segments, 237 (99.6%) were graded as acceptable, and 1 (0.4%) was graded as minor errors, whereas none were graded as unreliable.

### Annotation Agreement Assessment

In the supplementary annotation verification analysis, 150 randomly sampled ECG segments from the in-house dataset were reassessed, including 50 non-AF segments, 50 AF segments, and 50 noise segments. Agreement with the original study annotations was high for both the reevaluation by the original annotator and the independent review by an additional cardiologist. Cohen κ was 0.91 for the comparison between the original annotations and the reevaluation by the original annotator and 0.95 for the comparison between the original annotations and the independent cardiologist, with corresponding agreement percentages of 94% and 96.7%, respectively. Detailed pairwise agreement (Cohen κ) and class-stratified agreement by label are provided in Table S4 in Multimedia Appendix 2.

### Performance of the 2D-CNN Model in Internal Validation

**Figure 5.**
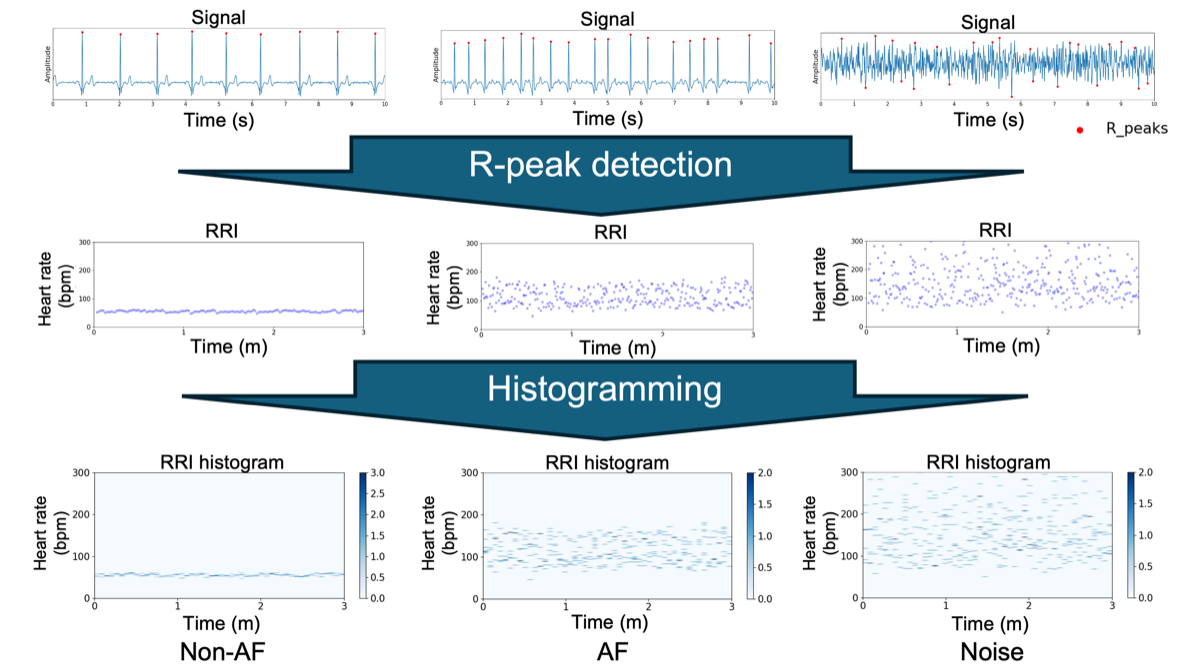
Generation of R-R interval (RRI) histogram images and representative examples used for 3-class classification. Schematic of the transformation from the RRI time series to 2D histogram images, together with representative examples of non–atrial fibrillation (AF), AF, and noise windows. The noise example represents an actual artifact-contaminated waveform recorded using the garment-type wearable Holter ECG device and the corresponding RRI-derived heart rate histogram, not simulated Gaussian noise.

The RRI histogram images were generated for each window length. Representative images of each class are shown in Figure 5. The proportion of candidate training windows excluded because they contained both AF and non-AF labels was less than 0.1% of all candidate windows. Exact ties requiring the application of the predefined tie-breaking rule were uncommon in the test data label aggregation process, occurring in 0% of 1.5-minute windows, 0.4% of 3-minute windows, and 0.3% of 6-minute windows. These low frequencies indicate that the tie-breaking rule had minimal influence on the overall evaluation. The mean performance metrics across the 5 cross-validation folds for each window length are presented in Table 2, and the detailed class-wise metrics, calculated in a one-versus-rest manner for the non-AF, AF, and noise classes, are provided in Table S5 in Multimedia Appendix 2.

**Table 2.** Window-level performance metrics across the 5 cross-validation folds for the 1.5-, 3-, and 6-min models in the internal validation cohort.

Window	1.5 min, mean (SD)	3 min, mean (SD)	6 min, mean (SD)
Accuracy	0.9656 (0.0116)	0.9664 (0.0110)	0.9610 (0.0106)
Macro *F*_1_-score	0.9633 (0.0115)	0.9641 (0.0109)	0.9582 (0.0120)
Macro AUROC^a^^,^^b^	0.9958 (0.0026)	0.9961 (0.0025)	0.9955 (0.0022)
AF^c^_Sensitivity	0.9675 (0.0318)	0.9666 (0.0268)	0.9489 (0.0418)
AF_Specificity	0.9800 (0.0090)	0.9817 (0.0081)	0.9835 (0.0037)
AF_PPV^d^	0.9558 (0.0220)	0.9591 (0.0214)	0.9636 (0.0109)
AF_NPV^e^	0.9859 (0.0135)	0.9849 (0.0112)	0.9761 (0.0187)

a Calculated using a one-versus-rest approach across the 3 classes.

b AUROC: area under the receiver operating characteristic curve.

c AF: atrial fibrillation.

d PPV: positive predictive value.

e NPV: negative predictive value.

The 1.5- and 3-minute windows achieved similarly high overall performance, with mean (SD) cross-validation accuracies of 96.6% (SD 1.2) for both models, macro *F*_1_-scores of 96.3% (SD 1.2) and 96.4% (SD 1.1), and macro AUROCs of 99.6% (SD 0.3) and 99.6% (SD 0.3), respectively. For the AF class, the 3-minute model showed slightly better balance than the 1.5-minute model, with comparable sensitivity of 96.7% (SD 2.7) versus 96.7% (SD 3.2), and slightly higher specificity of 98.2% (SD 0.8) versus 98.0% (SD 0.9) and PPV of 95.9% (SD 2.1) versus 95.6% (SD 2.2). In contrast, the 6-minute model showed slightly lower overall accuracy of 96.1% (SD 1.1), macro F1-score of 95.8% (SD 1.2), and AF sensitivity of 94.9% (SD 4.2), although AF specificity remained high at 98.4% (SD 0.4). Performance for the non-AF class was also consistently high across window lengths, indicating stable discrimination of AF from non-AF rhythms in the 3-class framework. Noise class performance appeared favorable in the internal validation, although its interpretation should remain cautious in light of the lower external noise sensitivity discussed in the following paragraph. Receiver operating characteristic curves for each window length are shown in Figure S3 in Multimedia Appendix 1.

In an exploratory post hoc subgroup analysis by garment type using pooled out-of-fold predictions from the 3-minute model, performance was similar between shirt-type and belt-type recordings. Accuracy was 96.8% in shirt-type recordings and 96.5% in belt-type recordings, and AF sensitivity was 97.4 and 96.7, respectively. However, the shirt-type subgroup included fewer patients and showed substantial class imbalance; therefore, definitive conclusions regarding garment-specific performance differences cannot be drawn. The detailed subgroup results are provided in Table S6 in Multimedia Appendix 2.

### Performance of the 2D-CNN Model in External Validation

The final DL models, trained using the 117-patient institutional dataset, were evaluated using the MIT-BIH AFDB to assess their generalizability. In the primary external validation, AFL-annotated intervals were excluded to align the test-label definition with the training task, in which AT/AFL episodes had been excluded. The results are summarized in Table 3.

**Table 3.** Window-level performance metrics in the primary external validation analysis using the MIT-BIH^a^ Atrial Fibrillation Database (AFDB).

MIT-BIH AFDB	1.5-min window model	3-min window model	6-min window model
Accuracy	0.9659	0.9731	0.9663
Non-AF^b^_Sensitivity	0.9836	0.9774	0.9805
AF_Sensitivity	0.9416	0.9695	0.9469
Noise_Sensitivity	0.7959	0.7000	0.8000
Non-AF_Specificity	0.9448	0.9676	0.9464
AF_Specificity	0.9864	0.9772	0.9814
Noise_Specificity	0.9963	0.9998	0.9991

a MIT-BIH: Massachusetts Institute of Technology–Beth Israel Hospital

b AF: atrial fibrillation.

The external validation dataset consisted of 23 independent AFDB recordings (approximately 230 h in total). As the AFDB did not originally include an explicit noise label, visually uninterpretable windows were additionally annotated as noise. The resulting reference-label distribution was imbalanced across all window length settings: for the 1.5-minute setting, 5549 non-AF (59.7%), 3701 AF (39.8%), and 49 noise (0.5%) windows; for the 3-minute setting, 2785 non-AF (60%), 1834 AF (39.5%), and 20 noise (0.5%) windows; and for the 6-minute setting, 1385 non-AF (59.8%), 922 AF (39.8%), and 10 noise (0.4%) windows.

**Figure 6.**
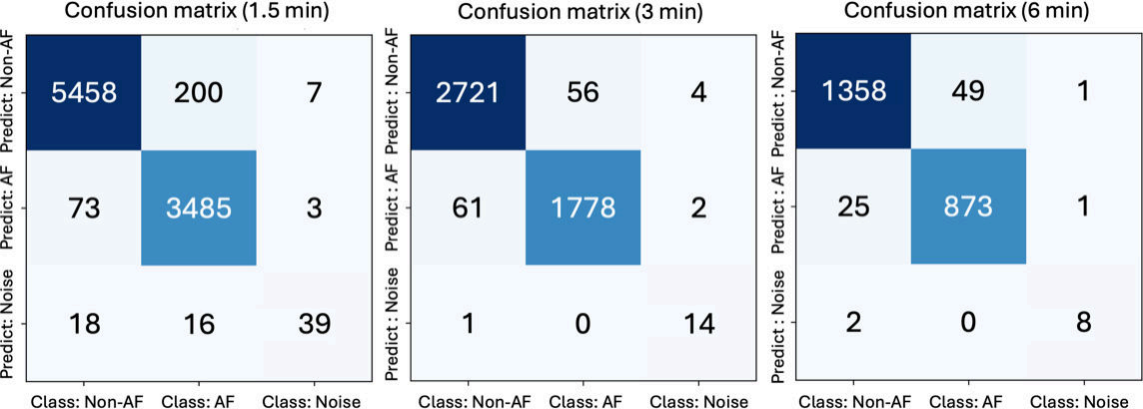
Confusion matrices for external validation of the 3-class classification task in the Massachusetts Institute of Technology–Beth Israel Hospital (MIT-BIH) Atrial Fibrillation Database (AFDB). Confusion matrices for the primary external validation analysis using 23 independent MIT-BIH AFDB recordings, with atrial flutter–annotated intervals excluded to match the institutional training task. The x-axis indicates reference labels, and the y-axis indicates model predictions for non–atrial fibrillation (AF), AF, and noise across the evaluated window lengths.

In the AFDB, the 3-minute model achieved the window-level accuracy of 97.3%, with an AF sensitivity of 96.9% and AF specificity of 97.7%. The 1.5-minute model achieved an overall accuracy of 96.6%, AF sensitivity of 94.2%, and AF specificity of 98.6%, representing a modest reduction in overall accuracy and AF sensitivity compared to the 3-minute model. The 6-minute model showed overall accuracy of 96.6%, AF sensitivity of 94.7%, and AF specificity of 98.1%, which were slightly lower than those of the 3-minute window. Accordingly, in the external dataset, the 3-minute window showed the highest overall accuracy and AF sensitivity while maintaining high AF specificity, although the differences across window lengths were modest and no formal statistical comparison was performed. Across all window lengths, however, sensitivity for the noise class was low: 79.6% (39/49), 70% (14/20), and 80% (8/10) for the 1.5-, 3-, and 6-minute models, respectively. This result should be interpreted cautiously because the number of noise-labeled windows in the external dataset was small and the noise characteristics in the AFDB may not have fully matched those in the institutional wearable dataset. Consistent with the class-wise metrics, the confusion matrices showed that AF and non-AF were generally well discriminated, whereas noise windows were relatively infrequent and more often misclassified than the other classes (Figure 6).

To further characterize the low noise sensitivity, we reviewed the false-negative noise windows in the 3-minute external validation analysis. Among the 20 noise-labeled windows, 6 were misclassified as non-AF or AF. Visual review of the corresponding ECG waveforms and RRI histograms suggested 2 common patterns. In 3 windows, the ECG waveform was visually nondiagnostic for rhythm interpretation, but the automatic R-peak detection algorithm generated structured RRI sequences, resulting in RRI histograms that did not show a typical scattered noise pattern. In the remaining 3 windows, visually noisy ECG portions were mixed with analyzable rhythm portions within the same 3-minute window, leaving structured RRI features in the model input despite the window-level noise label. Representative examples are shown in Figure S4 in Multimedia Appendix 1.

**Figure 7.**
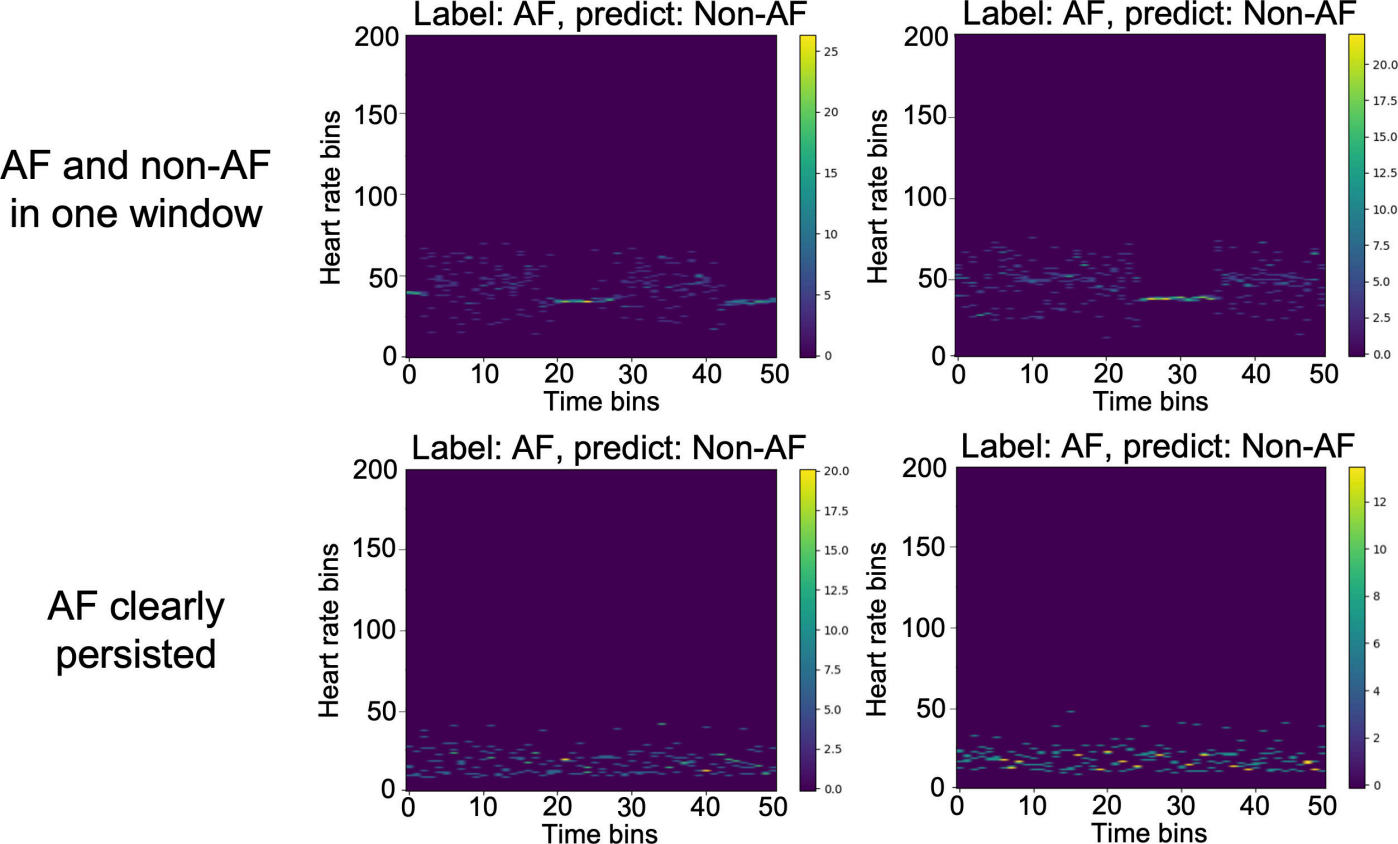
Representative misclassified atrial fibrillation (AF) windows in the primary external validation analysis. Representative examples from the 3-min model showing AF windows misclassified as non-AF in the MIT-BIH Atrial Fibrillation Database. The figure highlights cases in which AF and non-AF rhythms coexisted within a single window, as well as cases in which AF persisted beyond 3 min but was not detected.

We visually inspected the windows misclassified by the 3-minute model to characterize common patterns in the corresponding images. Among the AF windows misclassified as non-AF, 87.5% (49/56) corresponded to images in which AF and non-AF rhythms coexisted within the same 3-minute window, whereas windows in which AF clearly persisted beyond 3 minutes but was not detected accounted for 12.5% (7/56) of the cases. These findings indicate that most AF-to-non-AF misclassifications were associated with mixed-rhythm windows. Representative misclassified images are shown in Figure 7.

The results of the secondary analysis in which AFL intervals were mapped to the AF class are provided in Table S7 in Multimedia Appendix 2. Across all window lengths, performance in this secondary analysis was slightly lower than in the primary AFL-excluded analysis; for example, for the 3-minute model, accuracy decreased from 97.3% to 96.1%, and AF sensitivity decreased from 96.9% to 93.3%.

### Evaluation of AF Burden

**Figure 8.**
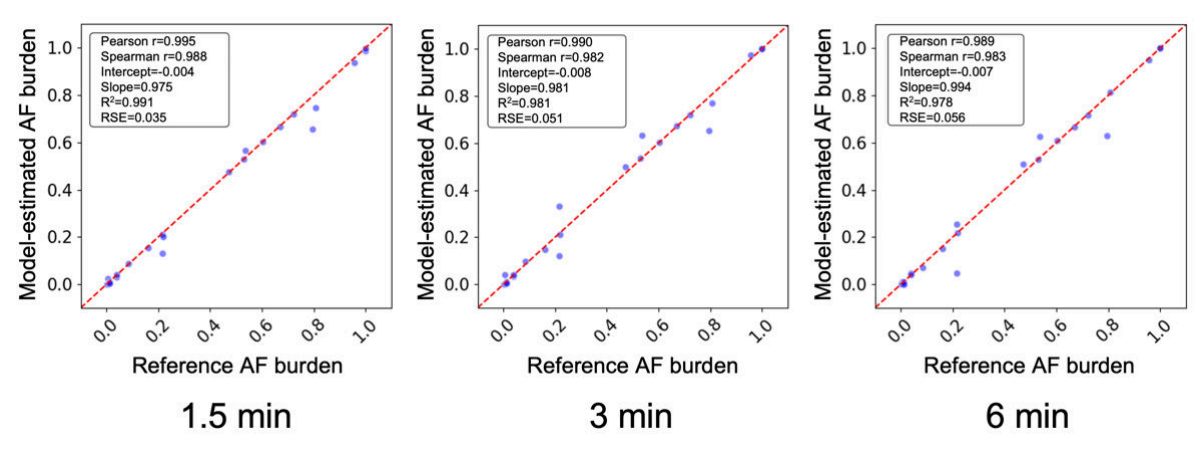
Patient-level atrial fibrillation (AF) burden correlation in the MIT-BIH Atrial Fibrillation Database external validation cohort. Scatter plot and regression line comparing reference AF burden with model-estimated AF burden for each of the 23 external validation recordings included in the primary atrial flutter–excluded analysis. AF burden was evaluated at the patient level by aggregating reference intervals and model predictions across each recording. RSE: residual standard error.

When AF burden was treated as a continuous patient-level variable derived from aggregated window-level classifications, the model showed a strong correlation with the reference AF burden across all 3 window length settings (Figure 8). Pearson correlation coefficients were 0.995 (*R*²=0.991), 0.991 (*R*²=0.982), and 0.989 (*R*²=0.979) for the 1.5-, 3-, and 6-minute models, respectively, and the corresponding Spearman rank correlation coefficients were 0.988, 0.982, and 0.979. Overall, patient-level AF burden correlation was high across all window lengths, with only small differences between models.

## Discussion

### Principal Findings

In this study, we developed a DL model that classifies RRI-derived 2D histogram images into non-AF, AF, and noise classes using a 2D-CNN. Internal and external validation suggested that the 3-minute window provided a clinically reasonable balance across the evaluated window lengths, although the differences were modest and should be interpreted as exploratory rather than definitive. In the external validation, the model also showed high correlation with the reference AF burden at the patient level. The contribution of this study lies not in the use of a novel network architecture, but in the application and evaluation of an RRI-based 3-class classification framework that explicitly incorporates noise in real-world garment-type wearable Holter ECG recordings.

### Comparison to Prior Work

For automated AF diagnosis, DL approaches can be broadly grouped into waveform-based models, RRI/heart rate variability (HRV)–based models, and multimodal frameworks that combine ECG-derived features with additional clinical information. Waveform-based models trained directly on raw ECG signals have shown high diagnostic performance in a variety of datasets, including residual network–based approaches, but their performance may depend substantially on recording format, window length, and dataset characteristics [18-22,31]. In contrast, RRI/HRV-based approaches focus on the beat-to-beat irregularity that is fundamental to AF and have been explored using transition-matrix features, entropy-based indices, multivariate classifiers, visual irregularity mapping, Lorenz/Poincaré-type representations, and image-based DL frameworks [4,23-25,32-34]. Recent multimodal work has further suggested that combining ECG with HRV and demographic information may improve AF detection performance in selected settings [35]. These studies provide useful context for the present findings, but direct numerical comparison should be interpreted cautiously because the underlying datasets, recording durations, input modalities, and intended use cases differ substantially. In this context, this study does not seek novelty in the network architecture itself; rather, its contribution lies in evaluating an RRI-based, 3-class, noise-aware framework in long-term garment-type wearable Holter ECG recordings, a setting in which noisy and clinically heterogeneous data are common. By converting the RRI time series into histogram images, this approach preserves AF-related irregularity patterns in a form suitable for CNN-based analysis while remaining conceptually closer to established HRV-based rhythm assessment than to raw waveform classification. As RRI-based representations use interval-derived information rather than full raw waveform input, converting an RRI time series into 2D images for CNN-based analysis may provide a conceptually simple and practical framework for AF screening in noisy wearable ECG recordings.

With a single-lead Holter or wearable ECG, reliably identifying P waves and fibrillatory waves (f waves), which are important for conventional AF diagnosis, can be challenging in the presence of noise. This problem is particularly pronounced in garment-type devices (eg, shirt- or belt-type systems), where artifacts arising from body movements and variable electrode contacts are common [8,36]. Garment-type Holter systems are expected to reduce the skin burden associated with adhesive electrodes, but they may be associated with more frequent noise contamination because of motion artifacts and variable electrode contact. Compared to curated public databases, real-world long-term Holter recordings contain a greater proportion of segments affected by noise artifacts. If such noise is not adequately identified, model performance, particularly specificity, may deteriorate, leading to an increased number of false positives [37]. RRI-based analysis offers a practical advantage in this setting; provided that R-peaks are accurately detected, it may be less dependent on raw waveform morphology than waveform-based approaches, making it attractive for handling noisy wearable ECG recordings [38].

To better handle noise-contaminated wearable ECG data, we implemented two additional strategies: (1) histogram binning of the RRI time series and (2) explicit annotation and learning of noise as a separate class. Histogram binning may provide a degree of local aggregation, thereby reducing the effects of minor timing fluctuations or isolated R-peak detection errors [39]. In addition, by formulating the task as a 3-class problem (non-AF, AF, and noise), the model was designed to distinguish uninterpretable segments from AF and non-AF rhythms without requiring a separate preprocessing step to exclude noisy data in advance. This framework may be advantageous for real-world wearable Holter ECG recordings, in which noise contamination is often unavoidable. Several previous studies have reported higher classification accuracies after rigorously excluding noisy segments from analysis [4,34,40]. However, such approaches may be less applicable to daily wearable monitoring, where preprocessing and manual cleaning are not always feasible. In this study, the AF diagnostic performance remained high despite the inclusion of noise-containing recordings, suggesting that this 3-class noise-aware framework is practical for AF screening in garment-type wearable ECG monitoring.

At the same time, noise sensitivity was substantially lower in the external validation than in the internal validation. One possible explanation is the very small proportion of noise-labeled segments in the external dataset, which makes sensitivity estimates unstable. Another possibility is a domain shift in noise characteristics: many noise segments in the institutional wearable dataset were associated with relatively prolonged signal degradation, such as electrode detachment or sustained signal loss, whereas the noise segments additionally annotated in the AFDB were retrospectively identified as visually apparent artifacts in a public dataset that did not originally include an explicit noise label. Additional review of false-negative noise windows suggested that some visually nondiagnostic ECG segments still yielded structured RRI histograms after automatic R-peak detection, while others contained mixed noisy and analyzable ECG portions within the same window. Thus, waveform-level visual noise annotation did not always correspond to degradation of the RRI-based feature representation used by the model, limiting the generalizability of noise classification across datasets. Although lower noise sensitivity should be regarded as a limitation, the consistently high noise specificity indicates that the model rarely assigned analyzable AF or non-AF windows to the noise class. This may be relevant in clinical review workflows, where excessive exclusion of interpretable rhythm segments could also be undesirable. The noise class in the present framework should therefore be interpreted as an operational class to avoid forcing unreliable windows into AF or non-AF categories, rather than as a universal artifact detector. Future implementations may benefit from complementary signal quality assessment, wear status detection, or preprocessing procedures.

The choice of the analysis window length for AF detection must be aligned with the model performance and the clinical significance of AF episodes. Previous studies using cardiac implantable electronic devices have suggested that stroke risk may increase when atrial high-rate episodes persist for ≥6 minutes, and 6 minutes have therefore been proposed as a clinically relevant threshold [28,29]. In this study, considering this evidence, we evaluated window lengths of 1.5, 3, and 6 minutes. Both internal and external validations suggested that the 3-minute window provided a clinically reasonable balance between AF sensitivity and specificity. However, the differences across window lengths were modest, and no formal statistical comparison was performed; therefore, this interpretation should be regarded as exploratory rather than definitive. The 3-minute model showed a high patient-level correlation with the reference AF burden in the AFDB, with performance comparable to that reported in previous studies [4,41]. A plausible explanation for the slightly lower AF sensitivity of the 6-minute model is that longer windows are more likely to include mixed rhythm or transitional segments, which may dilute AF-related irregularity within a single window; however, the high patient-level AF burden correlation suggests that the practical impact of this effect on overall burden estimation was limited. These window-level classification metrics and patient-level AF burden estimates address complementary aspects of performance: the former reflects the model’s ability to classify individual ECG windows, whereas the latter reflects the clinical interpretability of aggregating those predictions over an entire recording. Although small differences in AF burden estimates may not necessarily alter patient management in every clinical context, this analysis was included to show that the aggregated model outputs remained clinically meaningful beyond segment-level classification accuracy alone. From a practical monitoring perspective, a 3-minute window also allows AF status to be updated at relatively short intervals while still permitting consecutive-window aggregation to identify clinically relevant sustained episodes. Collectively, these findings indicate that a 3-minute RRI-based model may provide a practical balance for AF screening and AF burden assessment in ambulatory long-term Holter monitoring while allowing consecutive-window aggregation to support the detection of sustained AF episodes.

### Future Directions

As the present model was specifically designed to detect AF on the basis of RRI irregularity, AT/AFL episodes were excluded from the primary training and validation framework. AT/AFL often exhibit relatively regular RRIs and therefore do not consistently share the irregular rhythm pattern that characterizes AF, making them difficult to detect reliably using an RRI irregularity–based approach alone [26]. In a secondary analysis, in which AFL was grouped with the AF class for broader clinical categorization, diagnostic performance decreased, further supporting the limited suitability of the present RRI-based framework for identifying AT/AFL as AF. Therefore, future studies should explore a 2-stage algorithm in which RRI-based screening is followed by a detailed waveform-based analysis to identify AT/AFL among segments classified as non-AF. Incorporating additional features, such as waveform morphology and periodicity measures, may help construct a more comprehensive framework for diagnosing supraventricular arrhythmias using wearable ECG devices [42].

As the demand for AI-based diagnosis from single-lead ECG data increases, AF detection algorithms embedded in standard smartwatches still face limitations, including “unclassifiable” recordings and suboptimal sensitivity and specificity [43,44]. Conversely, the direct application of DL to smartwatch ECG waveforms can yield a performance that markedly surpasses that of built-in algorithms, suggesting that the potential for AI applications in wearable ECG will continue to grow. Within this context, the RRI-based 2D-CNN model developed in this study appears to be a promising AF screening approach that combines noise-aware design with potential practical applicability in prolonged high noise settings, such as garment-type wearable Holter monitoring. Our findings may contribute to the future implementation of garment-type wearable diagnostic systems and the development of efficient AF screening tools in primary care and community-based health care settings.

### Limitations

Although this study demonstrated the effectiveness of an RRI-based automatic AF detection model using single-lead Holter ECG recordings, it has several limitations.

First, although the CNN-based DL model achieved high classification performance, its decision-making process relied on internal latent representations and was not directly interpretable at the individual-prediction level. This inherent “black-box” nature reflects the broader challenge of explainability in medical AI and underscores the need for complementary approaches, such as model-agnostic explanation techniques, if the system is to be used for clinical decision support.

Second, because AT/AFL episodes and paced rhythms were excluded from the primary training and validation framework according to the predefined task definition, the present model was not designed or evaluated as a general classifier of supraventricular tachyarrhythmias or paced ECG recordings. In particular, pacemakers may artificially regulate RRIs and thereby alter the RRI irregularity patterns on which the present algorithm depends. Accordingly, its applicability to AT/AFL detection and to recordings containing paced rhythms remains limited and should be addressed in future studies using dedicated datasets and complementary features beyond RRI irregularity alone.

Third, although internal validation was performed using institutional data and external validation was conducted using the AFDB, the model’s generalizability across different clinical settings, patient populations, recording conditions, and ECG devices remains uncertain. In particular, the institutional and external validation datasets were enriched for AF relative to a general screening population. As PPV and NPV are mathematically dependent on disease prevalence, the favorable predictive values observed in this study may not directly translate to lower-prevalence screening settings; in particular, the PPV may be lower when the algorithm is applied to populations in which the true prevalence of AF is low. Noise characteristics can also vary substantially among wearable devices and datasets, and this may partly explain the markedly lower noise sensitivity observed in the external validation. Future studies should determine whether the same model, or a suitably adapted version, can be applied across diverse hardware platforms, external noise conditions, and lower-prevalence screening populations. In addition, because windows containing both AF and non-AF labels were excluded from training to avoid ambiguous supervisory signals, the reported classification performance may be somewhat optimistic relative to more heterogeneous real-world recordings containing frequent rhythm transitions. The proportion of such excluded candidate training windows was less than 0.1% in the present dataset. Consistent with this low transition rate, the patient-level AF burden distribution was bimodal, with many patients showing either no AF or very high AF burden and relatively few patients showing intermediate AF burden. Therefore, patients with highly active PAF characterized by frequent transitions between AF and non-AF rhythms were likely underrepresented. Moreover, because window-level reference labels were assigned using a majority vote rule, short AF episodes occupying less than half of a given window could be underrepresented, particularly in longer windows such as the 6-minute setting. Thus, the present framework should be interpreted as a window-level AF screening and burden estimation approach rather than a definitive episode-level detector for very brief AF events.

Fourth, the proposed method depends on automatic R-peak detection because the model input was derived from RRI data. Potential sources of R-peak detection error include noise and artifacts in Holter or wearable ECG recordings, as well as limitations in the acquisition system. Although the device incorporated analog band limiting intended for antialiasing before 250 Hz ADC sampling, the available specifications did not indicate a sharp low-pass filter below 100 Hz; therefore, residual aliasing of high-frequency components cannot be completely excluded. To assess the practical reliability of the R-peak detection step, we performed a supplementary manual quality assessment using 300 randomly sampled 30-second ECG segments; however, this was not an exhaustive beat-level validation of all 2850 hours of institutional recordings. In the external validation, manually annotated noise segments in the AFDB may not have fully reproduced the sustained mechanical artifacts encountered in garment-type wearable ECG recordings, and waveform-level visual noise labels may not always correspond to degradation of RRI-based feature representations. These factors may have contributed to the low noise sensitivity observed in the external validation. In addition, because the RRI-derived histogram was limited to a predefined heart rate range of 30 to 400 beats per minute, RRIs longer than 2.0 seconds were not explicitly represented as prolonged-pause features. Thus, clinically significant pauses, such as those observed in tachy-brady syndrome, would require separate pause-detection logic or waveform-based review.

Fifth, although we performed a rudimentary semiquantitative review of ectopic burden in patients with 0% AF burden, this assessment was based on sampled non-AF ECG segments and was not a formal beat-by-beat adjudication of premature atrial contractions or premature ventricular contractions across the entire non-AF class. The review suggested that the non-AF recordings were not limited to exceptionally clean sinus rhythm, but the precise prevalence and distribution of ectopic beats in all non-AF windows remain unknown. As the present model relies entirely on RRI irregularity, frequent ectopy may mimic AF-like irregularity, and may affect the interpretation of the model’s true specificity.

### Conclusions

This study developed and evaluated an RRI-based AF screening approach in which RRI time series data extracted from single-lead garment-type Holter ECGs were transformed into 2D images for DL-based classification. A 2D-CNN model trained on these visualized RRI features demonstrated high classification accuracy in both internal and external validations, suggesting potential utility for AF screening in prolonged monitoring settings using recordings without paced rhythms; recordings containing paced rhythms should currently be considered outside the intended use of this RRI-based algorithm. The 3-class framework, which explicitly separated noise from AF and non-AF rhythms, may be useful for handling real-world garment-type recordings that contain uninterpretable segments. However, broader validation across more diverse patient populations, recording environments, and device types will be necessary before routine clinical deployment can be considered. Importantly, the real-world performance of this framework will depend on the reliability of R-peak detection and on device- and dataset-specific noise characteristics.

Future work should further evaluate real-time feasibility and explore integration with waveform-based approaches for other supraventricular arrhythmias, such as AT/AFL.

## Supplementary material

10.2196/91960 Multimedia Appendix 1 Supplementary figures illustrating RRI histogram representations, representative noise-labeled ECG segments, one-vs-rest ROC curves, and external-validation examples for the 3-class AF screening model.

10.2196/91960 Multimedia Appendix 2 Supplementary tables summarizing patient-level AF and noise burden, ectopic burden assessment, annotation agreement, detailed model performance metrics, subgroup analysis by garment type, and secondary external validation results.

## Abbreviations

1D-CNN: one-dimensional convolutional neural network
2D-CNN: two-dimensional convolutional neural network
ADC: analog-to-digital converter
AF: atrial fibrillation
AFDB: MIT-BIH Atrial Fibrillation Database
AFL: atrial flutter
AT: atrial tachycardia
AUROC: area under the receiver operating characteristic curve
DL: deep learning
ECG: electrocardiography
HRV: heart rate variability
JR: junctional rhythm
MIT-BIH: Massachusetts Institute of Technology - Beth Israel Hospital
NPV: negative predictive value
NR: normal rhythm
PAF: paroxysmal atrial fibrillation
PPV: positive predictive value
RRI: R-R interval

## References

[1] Joglar JA, Chung MK, Writing Committee Members (2024). 2023 ACC/AHA/ACCP/HRS Guideline for the Diagnosis and Management of Atrial Fibrillation: a report of the American College of Cardiology/American Heart Association Joint Committee on Clinical Practice Guidelines. J Am Coll Cardiol.

[2] Nogami A, Kurita T, Kusano K (2022). JCS/JHRS 2021 guideline focused update on non-pharmacotherapy of cardiac arrhythmias. J Arrhythm.

[3] Fiorina L, Maupain C, Gardella C (2022). Evaluation of an ambulatory ECG analysis platform using deep neural networks in routine clinical practice. J Am Heart Assoc.

[4] Zhang P, Lin F, Ma F (2023). Automatic screening of patients with atrial fibrillation from 24-h Holter recording using deep learning. Eur Heart J Digit Health.

[5] Naydenov S, Jekova I, Krasteva V (2023). Recognition of supraventricular arrhythmias in Holter ECG recordings by ECHOView color map: a case series study. J Cardiovasc Dev Dis.

[6] Rahman MM, Rivolta MW, Badilini F, Sassi R (2026). Uncertainty estimation of deep learning models for atrial fibrillation detection from Holter recordings: a benchmark study. Biomed Signal Process Control.

[7] Shrikanth Rao SK, Kolekar MH, Martis RJ (2023). Atrial fibrillation detection using Poincare geometry and heart beat intervals. Expert Syst.

[8] Machino T, Aonuma K, Maruo K (2023). Randomized crossover trial of 2-week Garment electrocardiogram with dry textile electrode to reveal instances of post-ablation recurrence of atrial fibrillation underdiagnosed during 24-hour Holter monitoring. PLOS ONE.

[9] Joseph Michael Jerard V, Thilagaraj M, Pandiaraj K, Easwaran M, Govindan P, Elamaran V, B MA (2021). Reconfigurable architectures with high-frequency noise suppression for wearable ECG devices. J Healthc Eng.

[10] Amami K, Yoshihisa A, Horikoshi Y (2022). Utility of a novel wearable electrode embedded in an undershirt for electrocardiogram monitoring and detection of arrhythmias. PLOS ONE.

[11] McKenna S, McCord N, Diven J (2024). Evaluating the impacts of digital ECG denoising on the interpretive capabilities of healthcare professionals. Eur Heart J Digit Health.

[12] Aminorroaya A, Dhingra LS, Pedroso AF (2025). Development and multinational validation of an ensemble deep learning algorithm for detecting and predicting structural heart disease using noisy single-lead electrocardiograms. Eur Heart J Digit Health.

[13] Khunte A, Sangha V, Oikonomou EK (2023). Detection of left ventricular systolic dysfunction from single-lead electrocardiography adapted for portable and wearable devices. NPJ Digit Med.

[14] Fleury Q, Dubois R, Christophle-Boulard S, Extramiana F, Maison-Blanche P (2024). A deep learning modular ECG approach for cardiologist assisted adjudication of atrial fibrillation and atrial flutter episodes. Heart Rhythm O2.

[15] Mitchell H, Rosario N, Hernandez C, Lipsitz SR, Levine DM (2023). Single-lead arrhythmia detection through machine learning: cross-sectional evaluation of a novel algorithm using real-world data. Open Heart.

[16] Clifford G, Liu C, Moody B AF classification from a short single lead ECG recording: the physionet computing in cardiology challenge 2017.

[17] Christov I, Krasteva V, Simova I, Neycheva T, Schmid R (2018). Ranking of the most reliable beat morphology and heart rate variability features for the detection of atrial fibrillation in short single-lead ECG. Physiol Meas.

[18] Si J, Bao Y, Chen F (2025). Research on atrial fibrillation diagnosis in electrocardiograms based on CLA-AF model. Eur Heart J Digit Health.

[19] Ma H, Xia L (2023). Atrial fibrillation detection algorithm based on graph convolution network. IEEE Access.

[20] Feng K, Fan Z (2022). A novel bidirectional LSTM network based on scale factor for atrial fibrillation signals classification. Biomed Signal Process Control.

[21] Salinas-Martínez R, de Bie J, Marzocchi N, Sandberg F (2021). Detection of brief episodes of atrial fibrillation based on electrocardiomatrix and convolutional neural network. Front Physiol.

[22] Tarabanis C, Koesmahargyo V, Tachmatzidis D (2025). Artificial intelligence-enabled sinus electrocardiograms for the detection of paroxysmal atrial fibrillation benchmarked against the CHARGE-AF score. Eur Heart J Digit Health.

[23] Fang B, Chen J, Liu Y (2023). Dual-channel neural network for atrial fibrillation detection from a single lead ECG wave. IEEE J Biomed Health Inform.

[24] Kisohara M, Masuda Y, Yuda E, Ueda N, Hayano J (2020). Optimal length of R-R interval segment window for Lorenz plot detection of paroxysmal atrial fibrillation by machine learning. Biomed Eng Online.

[25] Yao Y, Jia Y, Wu M (2024). Detection of atrial fibrillation using a nonlinear Lorenz Scattergram and deep learning in primary care. BMC Prim Care.

[26] Weinberg KM, Denes P, Kadish AH, Goldberger JJ (2008). Development and validation of diagnostic criteria for atrial flutter on the surface electrocardiogram. Ann Noninvasive Electrocardiol.

[27] Hamilton P Open source ECG analysis.

[28] Uittenbogaart SB, Lucassen WAM, van Etten-Jamaludin FS, de Groot JR, van Weert H (2018). Burden of atrial high-rate episodes and risk of stroke: a systematic review. EP Europace.

[29] Healey JS, Connolly SJ, Gold MR (2012). Subclinical atrial fibrillation and the risk of stroke. N Engl J Med.

[30] He K, Zhang X, Ren S, Sun J Deep residual learning for image recognition.

[31] Seo HC, Oh S, Kim H, Joo S (2021). ECG data dependency for atrial fibrillation detection based on residual networks. Sci Rep.

[32] Patel S, Wang M, Guo J, Smith G, Chen C (2023). A study of R-R interval transition matrix features for machine learning algorithms in AFib detection. Sensors.

[33] Lown M, Brown M, Brown C (2020). Machine learning detection of atrial fibrillation using wearable technology. PLOS ONE.

[34] Krasteva V, Stoyanov T, Naydenov S, Schmid R, Jekova I (2025). Detection of atrial fibrillation in holter ECG recordings by ECHOView images: a deep transfer learning study. Diagnostics.

[35] Rawshani A, Rawshani A, Smith G (2025). Integrating deep learning with ECG, heart rate variability and demographic data for improved detection of atrial fibrillation. Open Heart.

[36] Xintarakou A, Sousonis V, Asvestas D, Vardas PE, Tzeis S (2022). Remote cardiac rhythm monitoring in the era of smart wearables: present assets and future perspectives. Front Cardiovasc Med.

[37] Burykin A, Costa MD, Citi L, Goldberger AL (2014). Dynamical density delay maps: simple, new method for visualising the behaviour of complex systems. BMC Med Inform Decis Mak.

[38] Zhao L, Liu C, Wei S, Shen Q, Zhou F, Li J (2018). A new entropy-based atrial fibrillation detection method for scanning wearable ECG recordings. Entropy.

[39] Jin X, Hirakawa K (2012). Analysis and processing of pixel binning for color image sensor. EURASIP J Adv Signal Process.

[40] Kim JY, Kim KG, Tae Y (2022). An artificial intelligence algorithm with 24-h Holter monitoring for the identification of occult atrial fibrillation during sinus rhythm. Front Cardiovasc Med.

[41] Hennings E, Coslovsky M, Paladini RE (2023). Assessment of the atrial fibrillation burden in Holter electrocardiogram recordings using artificial intelligence. Cardiovasc Digit Health J.

[42] Domazetoski V, Gligoric G, Marinkovic M (2022). The influence of atrial flutter in automated detection of atrial arrhythmias - are we ready to go into clinical practice?". Comput Methods Programs Biomed.

[43] Fiorina L, Chemaly P, Cellier J (2024). Artificial intelligence-based electrocardiogram analysis improves atrial arrhythmia detection from a smartwatch electrocardiogram. Eur Heart J Digit Health.

[44] De Guio F, Rienstra M, Lillo-Castellano JM (2025). Enhanced detection of atrial fibrillation in single-lead electrocardiograms using a Cloud-based artificial intelligence platform. Heart Rhythm.

[45] GitHub.

