# Supplementary material for: R-R Interval Histogram-Based Deep Learning for 3-Class Atrial Fibrillation Screening in Garment-Type Wearable Holter Electrocardiogram Monitoring: Algorithm Development and Validation Study
Source: JMIR Med Inform. 2026 Jul 24;14:e91960. doi: 10.2196/91960 (PMC13402272; doi:10.2196/91960)
Supplement: Multimedia Appendix 1 [file medinform-v14-e91960-s001.pdf]

Figure S1. Enlarged representative R–R interval (RRI) histogram images for each class.

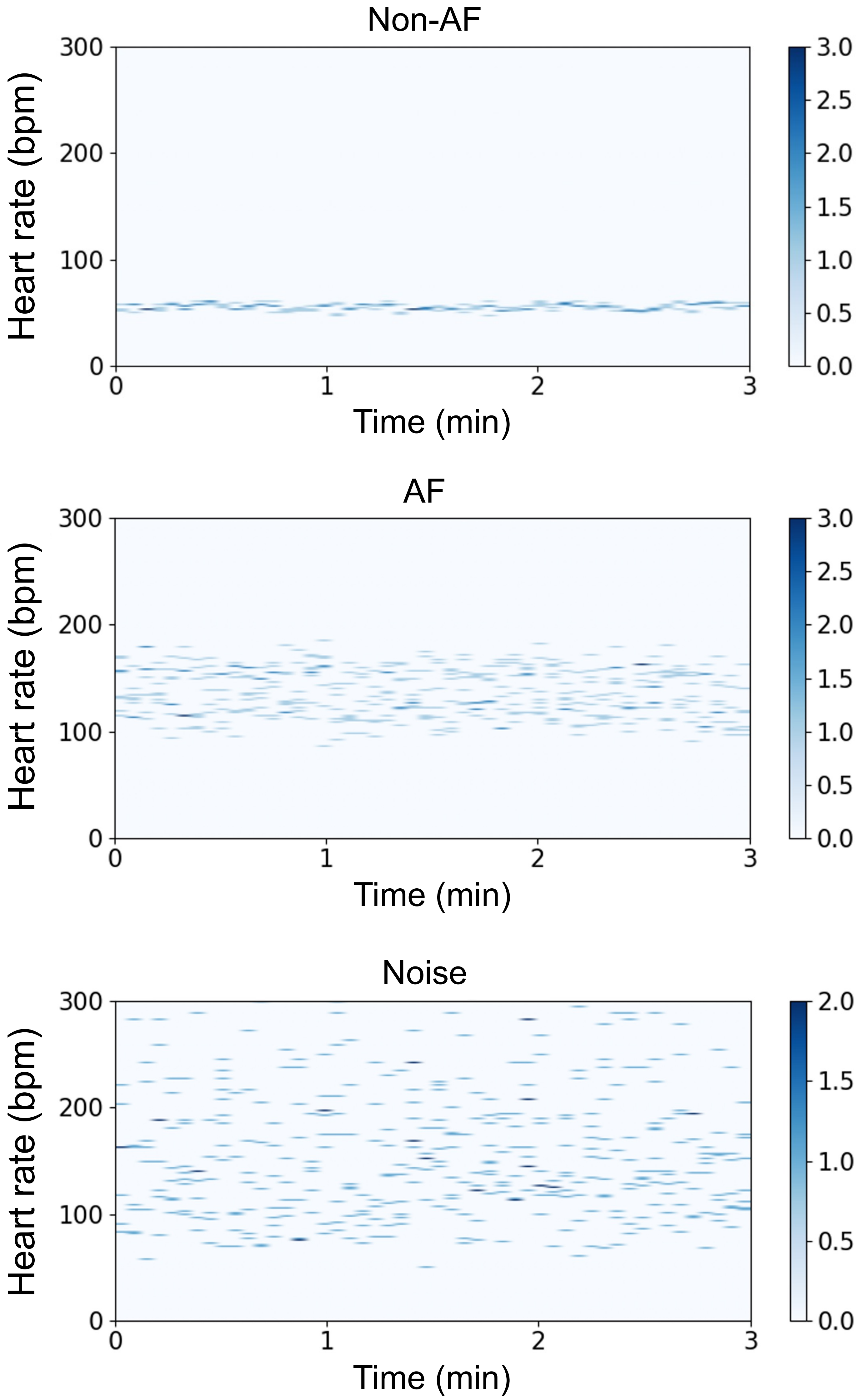

Representative two-dimensional RRI histogram images generated from a 3-minute analysis window are shown for each class (Non-AF, AF, and Noise). The x-axis indicates time (minutes) within the analysis window, and the y-axis indicates heart rate (beats per minute). Color intensity represents the binned count of RRI-derived heart rate values at each time point, illustrating the characteristic patterns used as inputs to the 2D-CNN model. Color scales are displayed separately for visual clarity. This enlarged version is provided to improve visual interpretability of the histogram format.
AF: atrial fibrillation; RRI: R–R interval.

Figure S2. Representative R-peak detection patterns in Noise-labeled ECG segments.

Type 1: Excessive false detections

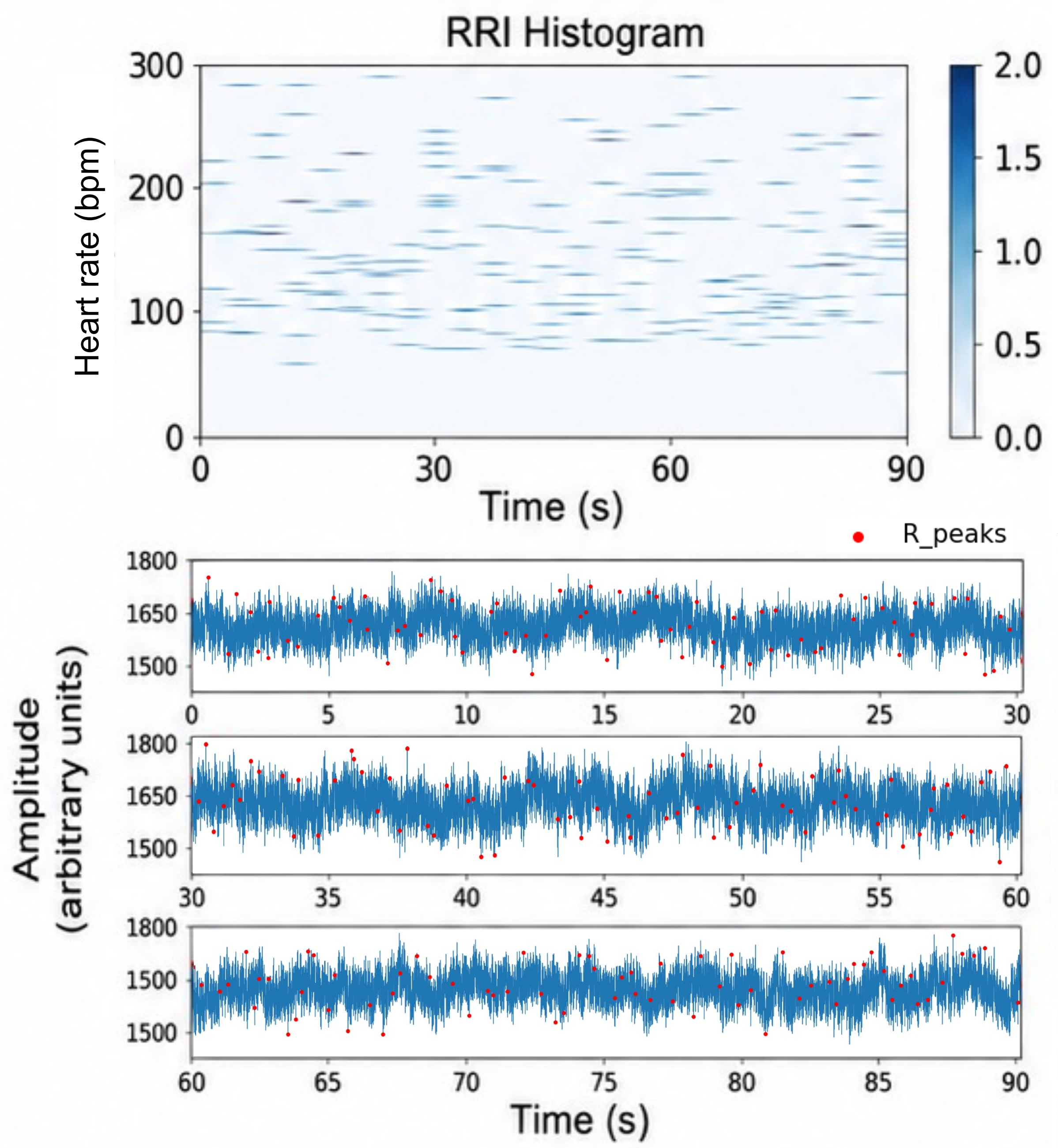

Type 2: Absent/insufficient detection

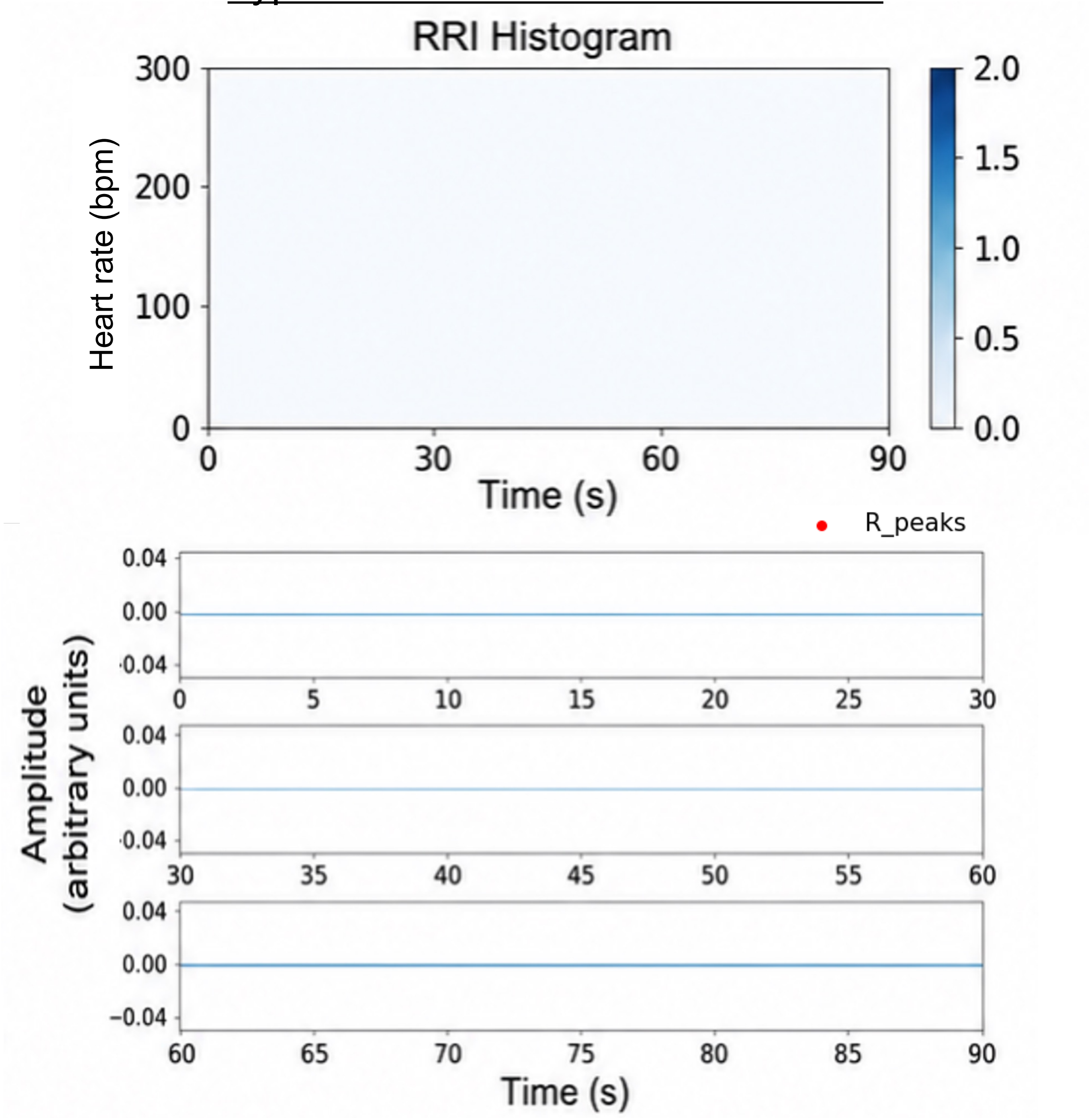

Type 3: Temporally heterogeneous artifact

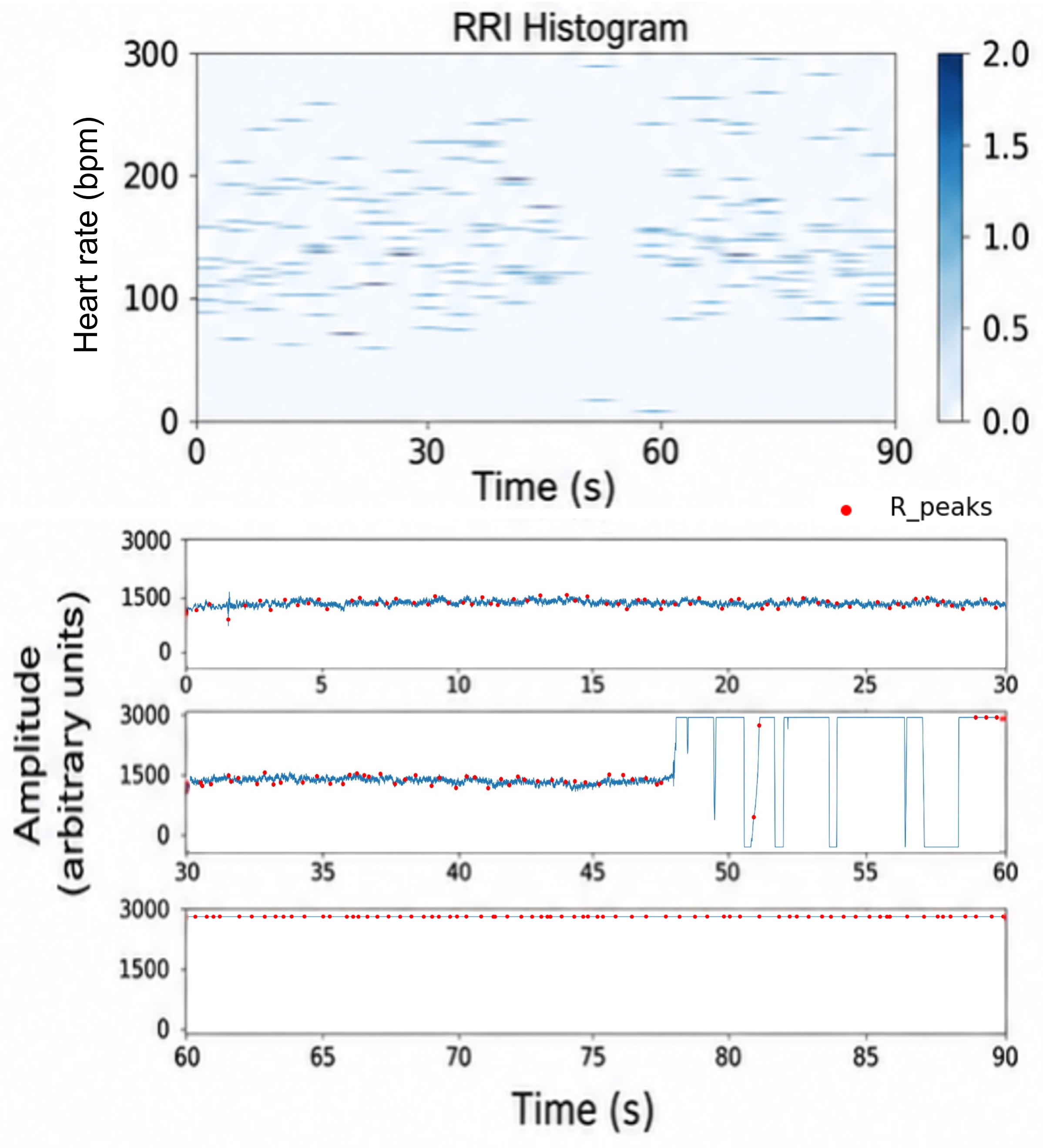

Real artifact-contaminated or signal-loss ECG waveforms recorded using the garment-type wearable Holter ECG device are shown with automatically detected R-peak positions and corresponding RRI-derived heart-rate histograms. For visual clarity, 1.5-min examples are shown. Noise-labeled segments showed heterogeneous R-peak detection behavior, including excessive false detections with physiologically implausibly short RR intervals, absent or insufficient R-peak detection resulting in an empty or sparse RRI-derived histogram, and temporally heterogeneous artifacts in which different failure modes, including excessive false detections, absent detections, and saturation-like false detections, coexisted within the same window. Artifact-related deflections or saturation-like signal changes could be falsely detected as R peaks. These representative examples illustrate that the Noise class was not defined by a single R-peak detection failure mode but by visually uninterpretable or non-diagnostic ECG quality.
ECG: electrocardiogram; RRI: R-R interval.

Figure S3. Out-of-fold ROC curves (one-vs-rest) by window length.

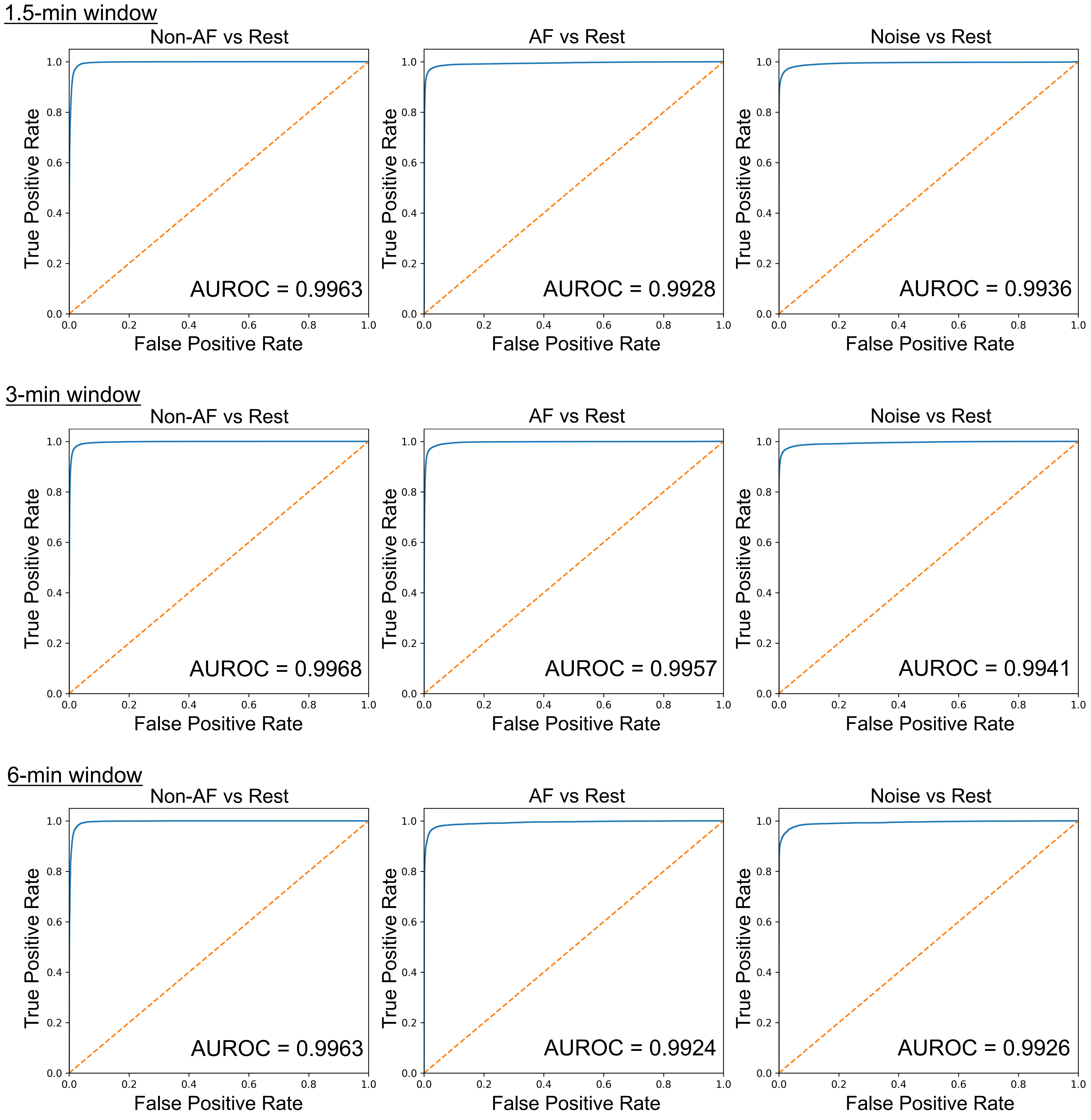

We evaluated discriminative performance using ROC curves and AUROC. ROC curves were generated from out-of-fold predictions obtained in five-fold inter-patient cross-validation. One-vs-rest ROC curves are shown for each class (Non-AF vs rest, AF vs rest, and Noise vs rest) at three analysis window lengths (1.5, 3, and 6 minutes). The corresponding AUROC values are displayed in each panel, demonstrating consistently high discrimination across classes and window lengths (all AUROCs ≥0.99).

ROC: receiver operating characteristic; AF: atrial fibrillation; AUROC: area under the ROC curve.

Figure S4. Representative false-negative Noise windows in the 3-min external validation analysis.

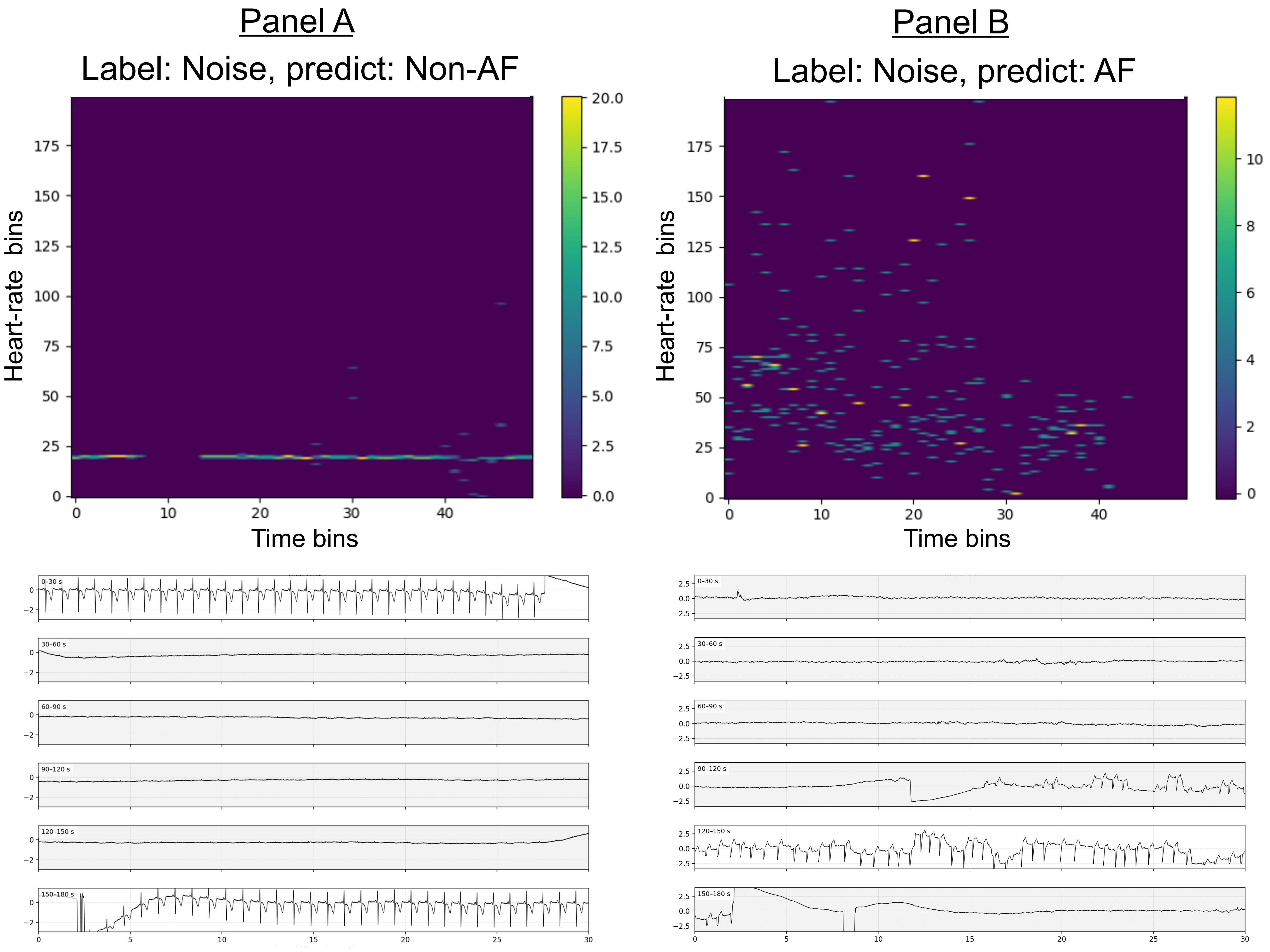

Representative examples of Noise-labeled 3-min windows that were misclassified in the external validation using the MIT-BIH Atrial Fibrillation Database. In each example, the upper panel shows the RRI-derived heart-rate histogram used as the model input, and the lower panels show the corresponding ECG waveform divided into 30-second rows. Background shading indicates the reference annotation at the waveform level: white, Non-AF; gray, Noise. Panel A shows a visually non-diagnostic ECG segment in which the automatic R-peak detection algorithm nevertheless generated a structured RRI sequence, leading to classification as Non-AF. Panel B shows a Noise-labeled window containing residual analyzable ECG portions, which generated irregular RRI features and led to classification as AF. These examples illustrate that waveform-level visual Noise annotation and RRI-based feature representation may not always be concordant.

RRI: R-R interval; ECG: electrocardiography; AF: atrial fibrillation.
