# Supplementary material for: R-R Interval Histogram-Based Deep Learning for 3-Class Atrial Fibrillation Screening in Garment-Type Wearable Holter Electrocardiogram Monitoring: Algorithm Development and Validation Study
Source: JMIR Med Inform. 2026 Jul 24;14:e91960. doi: 10.2196/91960 (PMC13402272; doi:10.2196/91960)
Supplement: Multimedia Appendix 2 [file medinform-v14-e91960-s002.docx]

**Table S1.** Distribution of patient-level atrial fibrillation burden in the institutional cohort.

| AF burden category | Patients, n (%) |
| --- | --- |
| 0% | 53 (45.3) |
| >0% to <10% | 3 (2.6) |
| 10% to <20% | 4 (3.4) |
| 20% to <30% | 3 (2.6) |
| 30% to <50% | 3 (2.6) |
| 50% to <80% | 2 (1.7) |
| ≥80% | 49 (41.9) |
| Total | 117 (100.0) |

Distribution of patient-level AF burden in the institutional cohort. AF burden was calculated as AF / (AF + Non-AF) using the original 10-second annotations; Noise-labeled intervals were excluded from the denominator. The distribution was bimodal, with many patients having either 0% or ≥80% AF burden, suggesting that patients with intermediate AF burden and frequent AF/Non-AF transitions were relatively underrepresented. AF: atrial fibrillation.

**Table S2.** Semiquantitative ectopic burden among patients with 0% AF burden.

| Estimated ectopic burden | Patients, n (%) |
| --- | --- |
| <1% | 36 (67.9) |
| 1% to <5% | 8 (15.1) |
| 5% to <10% | 3 (5.7) |
| ≥10% | 6 (11.3) |
| Total | 53 (100.0) |

Ectopic burden was estimated by semiquantitative visual review of patients with an AF burden of 0%. For each patient, six 3-min pure Non-AF ECG segments were sampled and reviewed together with R-peak markers and RRI plots. Estimated ectopic burden was calculated as the number of premature beat-like ectopic events divided by the number of detected R peaks in the reviewed segments. PACs and PVCs were not distinguished. This assessment was intended as a rudimentary estimate of ectopy-related RRI irregularity and was not a formal beat-by-beat adjudication across the entire Non-AF class. AF: atrial fibrillation; ECG: electrocardiogram; RRI: R-R interval; PAC: premature atrial complex; PVC: premature ventricular complex.

**Table S3.** Patient-level Noise burden in the institutional garment-type wearable Holter ECG cohort.

| Characteristics | Value |
| --- | --- |
| Total Noise-labeled intervals | 249,285 intervals |
| Total Noise-labeled duration | 692.5 hours |
| Median Noise burden (%) | 17.6 |
| IQR (%) | 7.0 – 36.0 |

| Noise burden category | Patients, n (%) |
| --- | --- |
| 0% | 2 (1.7) |
| >0% to <25% | 70 (59.8) |
| 25% to <50% | 30 (25.6) |
| 50% to <75% | 9 (7.7) |
| ≥75% | 6 (5.1) |

Noise burden was calculated as the proportion of Noise-labeled intervals among all annotated intervals for each patient. Total Noise-labeled duration was calculated by multiplying the number of Noise-labeled 10-second intervals by 10 seconds. ECG: electrocardiogram; IQR: interquartile range

**Table S4.** Supplementary annotation agreement assessment.

Part A. Pairwise agreement with the original study annotations and between reassessments

| Comparison | n | Agreement, n (%) | Cohen’s κ |
| --- | --- | --- | --- |
| Original vs Re-review | 150 | 141 (94.0) | 0.91 |
| Original vs Independent review | 150 | 145 (96.7) | 0.95 |
| Re-review vs Independent review | 150 | 144 (96.0) | 0.94 |

**Original**: original study annotations used in the main analysis

**Re-review**: repeat review of the sampled segments by the original annotator using the dedicated review program

**Independent review**: review of the sampled segments by an independent cardiologist

Part B. Agreement stratified by the label used for stratification

| Stratification label | Comparison | n | Agreement,  n (%) |
| --- | --- | --- | --- |
| Non-AF | Original vs Re-review | 50 | 46 (92.0) |
| AF | Original vs Re-review | 50 | 46 (92.0) |
| Noise | Original vs Re-review | 50 | 49 (98.0) |
| Non-AF | Original vs Independent review | 50 | 47 (94.0) |
| AF | Original vs Independent review | 50 | 49 (98.0) |
| Noise | Original vs Independent review | 50 | 49 (98.0) |
| Non-AF | Re-review vs Independent review | 51 | 47 (92.2) |
| AF | Re-review vs Independent review | 48 | 46 (95.8) |
| Noise | Re-review vs Independent review | 51 | 51 (100.0) |

**Note:** For “Original vs Re-review” and “Original vs Independent review,” stratification was based on the original study label. For “Re-review vs Independent review,” stratification was based on the re-review label.

AF: atrial fibrillation.

**Table S5.** Detailed metrics calculated in a one-versus-rest manner for the Non-AF, AF, and Noise classes.

| 1.5-min window | Non-AF | AF | Noise |
| --- | --- | --- | --- |
| Sensitivity | 0.9817 ± 0.0159 | 0.9675 ± 0.0318 | 0.9322 ± 0.0143 |
| Specificity | 0.9719 ± 0.0174 | 0.9800 ± 0.0090 | 0.9929 ± 0.0049 |
| PPV | 0.9668 ± 0.0182 | 0.9558 ± 0.0220 | 0.9784 ± 0.0120 |
| NPV | 0.9856 ± 0.0124 | 0.9859 ± 0.0135 | 0.9783 ± 0.0053 |
| F1-score | 0.9740 ± 0.0104 | 0.9611 ± 0.0159 | 0.9547 ± 0.0102 |
| AUROC | 0.9973 ± 0.0016 | 0.9958 ± 0.0046 | 0.9944 ± 0.0036 |

| 3-min window | Non-AF | AF | Noise |
| --- | --- | --- | --- |
| Sensitivity | 0.9868 ± 0.0129 | 0.9666 ± 0.0268 | 0.9294 ± 0.0109 |
| Specificity | 0.9687 ± 0.0124 | 0.9817 ± 0.0081 | 0.9951 ± 0.0024 |
| PPV | 0.9625 ± 0.0127 | 0.9591 ± 0.0214 | 0.9832 ± 0.0070 |
| NPV | 0.9881 ± 0.0123 | 0.9849 ± 0.0112 | 0.9778 ± 0.0053 |
| F1-score | 0.9744 ± 0.0098 | 0.9625 ± 0.0173 | 0.9555 ± 0.0069 |
| AUROC | 0.9970 ± 0.0028 | 0.9963 ± 0.0039 | 0.9948 ± 0.0023 |

| 6-min window | Non-AF | AF | Noise |
| --- | --- | --- | --- |
| Sensitivity | 0.9910 ± 0.0078 | 0.9489 ± 0.0418 | 0.9220 ± 0.0061 |
| Specificity | 0.9598 ± 0.0159 | 0.9835 ± 0.0037 | 0.9927 ± 0.0061 |
| PPV | 0.9526 ± 0.0162 | 0.9636 ± 0.0109 | 0.9750 ± 0.0230 |
| NPV | 0.9921 ± 0.0076 | 0.9761 ± 0.0187 | 0.9761 ± 0.0042 |
| F1-score | 0.9713 ± 0.0061 | 0.9557 ± 0.0212 | 0.9476 ± 0.0120 |
| AUROC | 0.9973 ± 0.0011 | 0.9957 ± 0.0044 | 0.9935 ± 0.0023 |

Values are reported as mean ± SD across the five cross-validation folds.

AF: atrial fibrillation; PPV: positive predictive value; NPV: negative predictive value;

AUROC: area under the receiver operating characteristic curve.

**Table S6.** Exploratory subgroup analysis of 3-min model performance by garment type using pooled out-of-fold predictions.

| Garment type | Shirt | Belt |
| --- | --- | --- |
| Patients | 25 | 92 |
| Windows | 15208 | 41761 |
| Non-AF_windows | 10941 | 14369 |
| AF_windows | 1890 | 16150 |
| Noise_windows | 2377 | 11242 |
| Accuracy | 0.9677 | 0.9654 |
| AF_Sensitivity | 0.9735 | 0.9672 |

Values were calculated using pooled out-of-fold predictions from the same five-fold cross-validation splits as the primary internal validation. The model was trained using mixed shirt- and belt-type recordings in each fold; only the test-fold predictions were stratified by garment type. Separate garment-specific models were not trained because the shirt-type subgroup had a limited sample size. AF sensitivity was calculated as true-positive AF windows divided by all reference AF windows in each garment-type subgroup. The subgroup analysis was exploratory and descriptive; no formal statistical comparison was performed. Interpretation is limited by the smaller number of shirt-type patients and the imbalanced distribution of Non-AF, AF, and Noise windows. AF: atrial fibrillation.

**Table S7.** Window-level performance metrics in the secondary external validation analysis in which AFL intervals were mapped to the AF class.

| MIT-BIH AFDB | 1.5-min window | 3-min window | 6-min window |
| --- | --- | --- | --- |
| Accuracy | 0.9551 | 0.9613 | 0.9568 |
| Non-AF_Sensitivity | 0.9889 | 0.9866 | 0.9832 |
| AF_Sensitivity | 0.9145 | 0.9329 | 0.9222 |
| Noise_Sensitivity | 0.5000 | 0.4545 | 0.6667 |
| Non-AF_Specificity | 0.9104 | 0.9252 | 0.9263 |
| AF_Specificity | 0.9889 | 0.9868 | 0.9834 |
| Noise_Specificity | 0.9986 | 0.9998 | 0.9970 |

Sensitivity and specificity were calculated in a one-vs-rest manner for each class.

AF: atrial fibrillation; AFL: atrial flutter.
